# Intraindividual Variability and Temporal Stability of Mid-Sleep on Free and Workdays

**DOI:** 10.1177/0748730420974842

**Published:** 2020-12-22

**Authors:** Anita Lenneis, Ahuti Das-Friebel, Henrik Singmann, Maris Teder-Laving, Sakari Lemola, Dieter Wolke, Nicole K. Y. Tang, Adrian von Mühlenen, Jüri Allik, Anu Realo

**Affiliations:** * Department of Psychology, University of Warwick, Coventry, UK; † Faculty of Brain Sciences, University College London, London, UK; ‡ Estonian Genome Center, University of Tartu, Tartu, Estonia; § Department of Psychology, Bielefeld University, Bielefeld, Germany; || Division of Health Sciences, Warwick Medical School, University of Warwick, Coventry, UK; ¶ Institute of Psychology, University of Tartu, Tartu, Estonia; # The Estonian Academy of Sciences, Tallinn, Estonia

**Keywords:** chronotype, mid-sleep, intraindividual variability, temporal stability, questionnaire validation

## Abstract

People differ in their sleep timings that are often referred to as a chronotype and can be operationalized as mid-sleep (midpoint between sleep onset and wake-up). The aims of the present studies were to examine intraindividual variability and longer-term temporal stability of mid-sleep on free and workdays, while also considering the effect of age. We used data from a 2-week experience sampling study of British university students (Study 1) and from a panel study of Estonian adults who filled in the Munich Chronotype Questionnaire twice up to 5 years apart (Study 2). Results of Study 1 showed that roughly 50% of the variance in daily mid-sleep scores across the 14-day period was attributed to intraindividual variability as indicated by the intraclass correlation coefficient. However, when the effect of free versus workdays was considered, the intraindividual variability in daily mid-sleep across 2 weeks was 0.71 the size of the interindividual variability. In Study 2, mid-sleep on free and workdays showed good levels of temporal stability—the retest correlations of mid-sleep on free and workdays were 0.66 and 0.58 when measured twice over a period of 0-1 to 5 years. The retest stability of mid-sleep scores on both free and workdays sharply increased from young adulthood and reached their peak when participants were in late 40 to early 50 years of age, indicating that age influences the stability of mid-sleep. Future long-term longitudinal studies are necessary to explore how age-related life circumstances and other possible factors may influence the intraindividual variability and temporal stability of mid-sleep.

Timing of sleep is an indicator of chronotype (Roenneberg et al., 2003) and is not identical with morningness-eveningness, which refers to one’s preference or natural inclination to go to and get out of bed (Horne and Östberg, 1976). The two are strongly related, yet distinct, constructs (Zavada et al., 2005). Chronotype, on one hand, refers to a time of day, namely, when an individual’s endogenous clock synchronizes (entrains) to the 24-h day (Roenneberg et al., 2004) and is often operationalized as mid-sleep, that is, midpoint between sleep onset and wake-up (Terman et al., 2001). Morningness-eveningness, on the other hand, is a diurnal preference for what time to go to and get out of bed, including at what time during the day an individual is most alert and can perform best (Horne and Östberg, 1976). People with an early or morning chronotype tend to wake up and to go to bed relatively early whereas those with a late or evening chronotype tend to sleep longer in the morning and to go to bed later. Chronotype has a strong biological and genetic basis with roughly half of the variance being attributed to heritability (see for example Hur, 2007; Koskenvuo et al., 2007; Barclay et al., 2010), whereas the remaining variance can be accredited to environmental and social factors such as the exposure to natural sunlight (Roenneberg et al., 2007), artificial light (Vetter et al., 2011), social and professional demands (Leonhard and Randler, 2009; Abbott et al., 2017), and personality (Lipnevich et al., 2017).

## Intraindividual Variability and Temporal Stability of Chronotype and Morningness-Eveningness

Despite sleep being an area of fast-developing interest due to its important role in people’s physical and mental health (e.g., Espie and Morin, 2012), the temporal stability of chronotype and morningness-eveningness across time has received far less attention. What is known from cross-sectional studies is that chronotype and morningness-eveningness vary with age: People are typically morning-oriented in childhood, become later chronotypes during adolescence with chronotype and morningness-eveningness peaking in lateness around the age of 18 to 20 years, and then gradually become earlier chronotypes again with increasing age, even more so from the age of 50 years (see Adan et al., 2012, for a review). However, cross-sectional studies do not allow us to examine intraindividual variability or change over time as any findings in cross-sectional studies may be due to cohort or period effects rather than age-related changes per se (Realo and Dobewall, 2011; Jacob and Ganguli, 2016). A study by Broms and colleagues (2014), for instance, examined morningness-eveningness across generations in the 1980s and 2000s and showed that there were less morning people and more evening people in the 2000s compared with the 1980s, indicating that the distribution of morningness-eveningness may be different across generations.

Most of the studies that have looked into the temporal stability of chronotype and morningness-eveningness have done so over relatively short periods of time in the course of validating and establishing test-retest reliabilities of chronotype and morningness-eveningness questionnaires. Supplemental Table S1.1 gives an overview of the previously conducted studies investigating test-retest correlations in sleep-time-based assessments of chronotype and preferential morningness-eveningness questionnaires over both shorter and longer periods of time. Generally speaking, the test-retest reliabilities of both types of questionnaires have been found to be quite high (i.e., range between *r* = 0.76 and 0.97) when the same participants were tested 1 to 4 months apart from each other. However, the test-retest reliabilities seem to decrease with longer time intervals. A longitudinal study by Urner et al. (2009), for instance, used actigraphy-based measures of mid-sleep on free days (MSF; chronotype) and mid-sleep on workdays (MSW) in a sample of 17- to 19-year-old students and found that the mid-sleep scores on free and workdays were correlated at *r* = 0.55 and −0.58 (*p* < 0.05) across two measurements 5 years apart, respectively. Also, when measuring the stability of diurnal type in a small subsample of the Older Finnish Twin Cohort, the findings of Koskenvuo and colleagues (2007) showed that there was a significant shift toward being a more morning person with increasing age across a 6-year period.

The intraindividual or within-person variability in chronotype, that is how an individual’s chronotype or mid-sleep fluctuates from one day to another (Mroczek et al., 2003), has rarely been examined. Such studies need intensive repeated measurement designs and hence are harder to conduct. A 14-day experience sampling study, for instance, that investigated night-to-night variability in various sleep behaviors and measures found that both participants with and without chronic insomnia had substantial night-to-night variations in quantitative and qualitative sleep diary measures as well as in actigraphy-based measures of sleep (Buysse et al., 2009). A related study found that adolescents with attention deficit hyperactivity disorder (ADHD) had greater intraindividual variability in their bedtimes and wake-up times than adolescents without ADHD (Langberg et al., 2019). These studies suggest that there might also be daily fluctuations in mid-sleep time points. When investigating biological markers of circadian rhythms during three 24-h sessions in the laboratory, Selmaoui and Touitou (2003) reported that participants had little daily intraindividual variability of cortisol circadian rhythm parameters and daily intraindividual variability in melatonin parameters. Differently from humans, however, there are many studies that have investigated the inter- and intraindividual variability of circadian rhythms in non- human animals. These studies have consistently shown that the interindividual variability of circadian markers is greater than the intraindividual variability (see for example Sharma, 1996; Refinetti and Piccione, 2005; Romeijn and Van Someren, 2011; Wassmer and Refinetti, 2019).

## The Aims of the Present Studies

To the best of our knowledge, none of the existing studies has examined the daily intraindividual variability in mid-sleep (i.e., a marker for chronotype) over a consecutive period of time in a natural setting. The handful of test-retest studies reporting on the temporal stability of chronotype thus far indicate that chronotype is remarkably stable across shorter time scales ranging from 2 weeks up to 1 year. However, only few studies have investigated the temporal stability of chronotype over longer periods of time and in different age groups. To fill these gaps in knowledge, we present two studies that extend previous research in several ways.

In Study 1, we examined the daily intraindividual variability in chronotype during a consecutive 14-day period among a sample of undergraduate students from the United Kingdom. More specifically, we were interested in the daily variability of the midpoint of sleep (i.e., midpoint between sleep onset and wake-up) which significantly predicts an individual’s dim light melatonin onset (DLMO) which is the gold standard for a circadian phase marker (Terman et al., 2001; Pandi-Perumal et al., 2007; Kantermann et al., 2015). Similar to previous studies (Urner et al., 2009; Zwart et al., 2018), we were not only interested in people’s MSF, which is often used to assess chronotype (Roenneberg et al., 2019), but also in people’s timing of MSW. In other words, we aimed to find out how large the intraindividual variability in daily mid-sleep scores is during the period of 14 days and to what extent the daily variability of mid-sleep is influenced by free and workdays. As mid-sleep is composed of sleep onset and wake-up time, we were also interested in the intraindividual variability of these two sleep variables and how they relate to mid-sleep.

We also examined the correspondence between the average daily estimates of mid-sleep both on free and workdays over the period of 2 weeks and (a) the recall-based estimates of mid-sleep as obtained with the Munich Chronotype Questionnaire (MCTQ; Roenneberg et al., 2003) as well as (b) actigraphy-derived estimates of mid-sleep. Research suggests that global retrospective measures which ask participants to report on their typical behavior over a certain period of time (e.g., the MCTQ asks about sleep behavior over the past 4 weeks) often fail to adequately characterize intraindividual variations over time and therefore, produce more biased estimates than momentary or daily assessments (Shiffman et al., 2008). Because studies have shown mixed agreement between objectively and subjectively assessed estimates of sleep (Lockley et al., 1999; Girschik et al., 2012), it is important to assess both types of sleep measurement. Thus, another aim of Study 1 was to validate the recall-based mid-sleep estimates on free and workdays as obtained with the MCTQ and the actigraphy-derived measures of mid-sleep against the daily mid-sleep assessments averaged over 2 weeks.

In Study 2, we investigated the temporal stability of chronotype over a period of 0-1 to 5 years and across different parts of the life span. As we reviewed above, both cross-sectional and a few longer-term longitudinal studies have shown that chronotype can—and mostly does—change across the life span. However, due to a limited age range of their samples, the existing studies have not been able to address the question of whether there is any systematic variation in the longer-term stability of chronotype across the life span. By using a sample of adults with an age range from 18 to 87 years who completed the MCTQ (Roenneberg et al., 2003) twice up to 5 years apart, we will explore the degree of rank-order stability of mid-sleep on both free and workdays across different stages of adulthood.

The pseudodata and scripts for Study 1 as well as the pseudodata and scripts for Study 2 can be downloaded at https://osf.io/e7q6y/?view_only=9a48becd4430488584f70eddb57cad82. For access to the real data, please apply at https://genomics.ut.ee/en/biobank.ee/data-access.

## Study 1: Intraindividual Variability in MID-SLEEP ON FREE AND WORKDAYS Across 2 Weeks

### Method

#### Participants

A total of 129 undergraduate students from a University in the United Kingdom signed up to take part in the study. We recruited them through the Student Mental Health and Resilience in Transition’s (SMaRT) online questionnaire where respondents indicated whether they were interested in participating in an experience sampling study. We also advertised the study on SONA—a system used across the University for advertising and booking into research studies. Of the 129 participants, 13 were not able to participate in the experience sampling study since they could not download the application on their phones that we used for the study. One participant dropped out after the first day of the study. We excluded four participants from the analyses since they did not provide enough information on their sleep to calculate average sleep scores.

As a result, the final sample consisted of 111 University undergraduates, 71 (63.96%) identified as female, 40 (36.04%) as male. Their mean age was 19.71 (SD = 1.58) years, ranging from 18 to 32 years. Of those, 104 (93.69%) had actigraphy data available. The dataset has been used in other studies (Das-Friebel et al., 2020) but it has not been used for the present purpose.

#### Procedure

Participants took part in a 2-week experience sampling study between October 2017 and March 2018. Due to a limited number of actigraphs, only a maximum of 25 participants could partake in the study at a time. Therefore, the data collection took place in five consecutive stages during the above-mentioned period.

During each stage of data collection, participants visited the laboratory twice, usually in groups of four to six. During the first introductory session, participants gave their informed consent and filled in an online questionnaire, which included sociodemographic questions as well as validated questionnaires about sleep quality, chronotype (i.e., the MCTQ), personality, diet, and affect. They then downloaded an application on their smartphones for the experience sampling study. To link the online questionnaire with the experience sampling data, the participants were given a randomly created unique identification code. At the end of the introductory session, participants received £5. The next morning, the experience sampling study started for the 2-week period. If participants had questions, they were advised to email the experimenters. After the 2-week period, participants came back to the laboratory for a debrief session. They were asked to fill in a short feedback questionnaire, return the actigraphs, and collect a remaining honorarium of up to £35 (depending on the number of surveys that they had filled in; one survey was equivalent to approximately £0.63).

The 14-day experience sampling study was conducted with Ilumivu’s mobile ecological momentary assessment app (mEMA; https://ilumivu.com/) which was compatible with both major mobile operating systems (i.e., Android OS and iOS). Participants were told that each day of the study they would receive two types of surveys—open and momentary surveys. The open survey launched every day at 0800 h and was left open for the next 24 h so that participants could complete the survey any time during the day. They received a reminder to fill it in at the start of the time window. It mainly consisted of questions about the previous day’s physical activity, diet, social media usage, and sleep but also about when participants woke up on the day of the survey. In addition, participants were randomly prompted to fill in a shorter momentary survey five times a day between 0800 h and 2200 h from Monday to Friday and between 1000 h and 2200 h Saturday and Sunday. At each prompt, participants had 20 min to fill in the survey and were advised to complete it as soon as they had received the prompt. During the momentary surveys, participants were asked about their current mood, well-being, what they were doing, and their social media usage. In the present study, only the data from the open survey will be analyzed.

Participants were also asked to wear an actigraph for the course of the study. Participants’ sleep was recorded with actigraphy the same night following the introduction session whereas the experience sampling study started the next day. We will use the daily actigraphy data for validation purposes.

#### Materials

##### MCTQ

Participants were asked to complete the English version of the MCTQ (Roenneberg et al., 2003) during the first introductory meeting before the 14-day experience sampling study began. The MCTQ consists of 17 items that ask about typical sleep behavior over the past 4 weeks separately for workdays and free days to take into account our modern lifestyle which often leads to a clash between biological and social clocks (Roenneberg et al., 2019). The MCTQ was designed to assess chronotype as biological phase of entrainment rather than preferences (Roenneberg et al., 2003; Roenneberg, 2015) and it measures chronotype as MSF after correcting for accumulated sleep debt over the week.

In our study, however, we did not correct MSF for sleep debt on workdays because MSF corrected for sleep debt on workdays is not defined as a daily score as it takes into account the sleep duration on free and workdays. It is also problematic to correct MSF for sleep debt on workdays when assessing the validity of the MCTQ scores as the ratio of free and workdays can differ considerably across participants while the correction uses 2 free days and 5 workdays (Kühnle, 2006). Furthermore, in a study by Kantermann and Burgess (2017), MSF correlated at *r* = 0.71 (*p* < 0.001) with the DLMO, which is currently seen as the gold standard for a circadian phase marker, whereas MSF corrected for sleep debt accumulated on workdays correlated at *r* = 0.68 (*p* < 0.001) with the DLMO, indicating that both measures assess chronotype or circadian rhythm equally well. We therefore refer to MSF as chronotype (MSF_MCTQ_) which we calculated as the midpoint between sleep onset (the time someone falls asleep) and wake-up time on free days. We also computed a midpoint of sleep on workdays (MSW_MCTQ_) in the same way as the MSF_MCTQ_. Lower scores of both MSF_MCTQ_ and MSW_MCTQ_ indicate a greater tendency to an early chronotype whereas higher scores indicate a disposition toward a later chronotype (Roenneberg et al., 2003). The MSF_MCTQ_ and MSW_MCTQ_ scores were correlated at *r* = 0.84 (*p* < 0.001). The MSF_MCTQ_ (M = 5.69; SD = 1.57) was significantly higher than MSW_MCTQ_ (M = 4.49; SD = 1.24), *t*(110) = 14.91, *p* < 0.001, indicating that people sleep later on free days than on workdays.

##### Measurement of mid-sleep in the experience sampling study

Since the MCTQ assesses mid-sleep retrospectively over the period of past 4 weeks, we had to adjust for this in the experience sampling study. More specifically, on each day of the experiment, participants were asked to answer the following questions: (a) “At what time did you get into bed last night?” (b) “At what time did you switch off the lights to fall asleep last night?” [*getting ready to fall asleep*]; (c) “How long did it take for you to fall asleep last night?” [*time it takes to fall asleep*]; (d) “At what time did you wake up this morning?” [*wake-up time*]; (e) “Did you use an alarm clock to wake up this morning?” (yes/no); and (f) “Is today a regular working/university day for you?” (yes/no). We used the last question to differentiate between MSW and MSF. To calculate the daily mid-sleep scores, we first had to calculate sleep onset for each day:

$$\begin{array}{l} sleep\;onset\;=\;getting\;ready\;to\;fall\;asleep\;\\ \;\;\;\;\;\;\;\;\;\;\;\;\;\;\;\;\;\;\;\;\;\;\;\;\;\;\;\;+time\;it\;takes\;to\;fall\;asleep. \end{array}$$

Next, we calculated the sleep duration for each day:

sleepduration=wake-uptime−sleeponset.

The daily mid-sleep scores were calculated using the following formula:

$$mid-sleep\;=\;sleep\;onset+\frac{sleep\;duration}{2}.$$

Overall, there were 1361 complete daily measurements of mid-sleep across participants out of 1547 possible measurements (104 participants × 14 days and 7 participants × 13 days due to technical issues), with the overall response rate being 87.98%. Across all participants, we further excluded 21 daily mid-sleep scores due to several reasons (Please refer to Supplemental Material S2). The average number of daily measurements of mid-sleep per participant was 12.25 (SD = 2.32), ranging from 3 to 14. The average daily mid-sleep score across the 14 days across all participants was 5.07 (SD = 1.27) which corresponds roughly to 0500 h.

Finally, we calculated average daily mid-sleep scores for free (MSF_ES_) and work (MSW_ES_) days across the 2-week study period. The free and workdays were set differently for each participant, depending on their answers to the question if the day on which they completed the daily measurement of sleep was a regular work-/university day for them or not. The average number of measurements for computing MSF_ES_ was 4.61 (SD = 2.14) and 7.45 (SD = 2.25) for MSW_ES_. The correlation between the MSF_ES_ and MSW_ES_ was *r* = 0.87, *p* < 0.001. The average daily mid-sleep score on free days (MSF_ES_; M = 5.26, SD = 1.48) was significantly higher than on workdays (MSW_ES_; M = 4.78, SD = 1.23), *t*(107) = 7.34, *p* < 0.001.

##### Actigraphy-derived measurements of mid-sleep

We used ActiGraph wGT3X-BT devices manufactured by ActiGraph to get objective estimates of mid-sleep. The actigraph recorded information about participants’ movements and activity using a three-axis accelerometer. Participants were not able to indicate on their actigraphs at what time they tried to fall asleep and gotten out of bed. Therefore, we used the information extracted from the sleep diaries (i.e., the daily open surveys of the experience sampling study) as anchoring points. We calculated the daily mid-sleep scores the same way as in the experience sampling study and then calculated an average score for MSF_ACT_ and MSW_ACT_. The two scores correlated at *r* = 0.77, *p* < 0.001, with each other. The actigraphy-derived average daily mid-sleep score on free days (MSF_ACT_; M = 5.65, SD = 1.51) was significantly higher than on workdays (MSW_ACT_; M = 4.86, SD = 1.18), *t*(100) = 8.44, *p* < 0.001.

### Results

#### Intraindividual Variability of Mid-sleep Over the Period of 14 Days

The main focus of Study 1 was to examine the amount of intraindividual variability in self-reported daily mid-sleep scores across a 2-week period. We performed the analyses using linear mixed models employing the afex package (Singmann et al., 2020) in R.

In a first model, we solely compared within- and between-individual variability across the full 2-week period. The model which had daily mid-sleep as the dependent variable only contained by-subject random intercepts and no further fixed effects. Results showed that intraindividual variability in daily mid-sleep scores (1.41; SD = 1.19) was approximately equal to the interindividual variability in daily mid-sleep scores (1.43; SD = 1.20). This can be expressed in terms of an intraclass correlation coefficient (ICC) according to which 50.46% of the variance can be explained by between-participant effects. However, the *ICC* is only well-defined for random-intercept-only models and thus we will not be using it in the following model.

Our first model did not allow for the possibility to examine systematic differences in daily mid-sleep scores between free and workdays. More specifically, one could imagine that people’s mid-sleep differs systematically between days they have to work versus days they do not have to work when they get up (e.g., compare Friday vs. Saturday in a standard European workweek)—we call this factor *workday today* (with two levels, workday vs. free day). In addition, another factor that might affect mid-sleep is whether the previous day was a free day versus workday (e.g., compare Saturday vs. Sunday in a standard European workweek)—we call this factor *workday yesterday* (with two levels, workday vs. free day).

We coded each day in our data on these two variables based on participants’ self-reports and estimated a second mixed model on the daily mid-sleep scores with fixed effects for factors *workday today* and *workday yesterday*, as well as for their interaction. For the random-effects structure, we initially started with the maximal random-effects structure justified by the design (Barr et al., 2013), which entailed by-participants random intercepts, by-participant random slopes for the two fixed effects and their interaction, as well as the correlation among the random slopes. Because this model showed a singular fit, we removed the random-slope for the interaction of the two fixed effects (this model still allowed us to retain the correlation among the remaining random-effect parameters).

**Figure 1.**
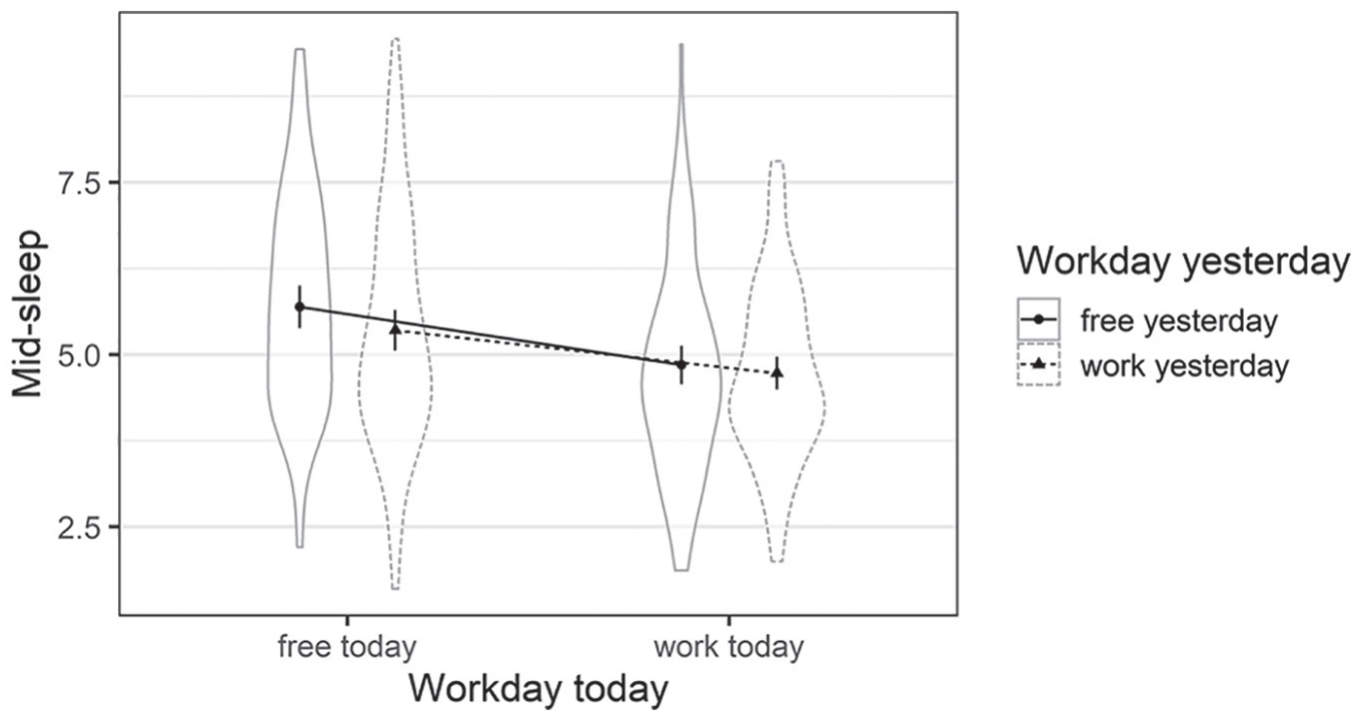
Violin plot of the mixed model depicting mid-sleep including work and free days today and yesterday. Mean scores across the sample are depicted as bold points together with their 95% confidence intervals. The violin plots depict per participant aggregated data (Study 1).

The test of the fixed effects was based on the Satterthwaite approximation, model predictions are displayed in Figure 1. We found significant effects for the two main effects, *workday today, F*(1, 101.55) = 68.10, *p* < 0.001, and *workday yesterday, F*(1, 101.87) = 8.62, *p* = 0.004. Daily mid-sleep scores were later on free days for both factors, that is, participants’ mid-sleep scores were later when they went to bed and got out of bed on a free day. Furthermore, the effect of *workday today* on daily mid-sleep scores was more pronounced than for *workday yesterday*, meaning that participants’ mid-sleep scores were mostly influenced by whether the day they woke up was a free or workday. The interaction between both factors did not reach significance, *F*(1, 972.21) = 2.46, *p* = 0.117.

Finally, we examined the intraindividual variance of daily mid-sleep scores in the second model when considering the effects of free versus workdays. When taking the above elaborated effect of free versus workday into account, the intraindividual variability (1.09; SD = 1.04) in daily mid-sleep scores was 0.71 the size of the interindividual variability (1.53; SD = 1.24). This indicates that when the effects of free versus workday are controlled for, the daily mid-sleep scores differ less within than between participants.

#### Intraindividual Variability of Bedtimes and Wake-up Times Over the Period of 14 Days

As mid-sleep is calculated as the midpoint between sleep onset and wake-up time, we were also interested to investigate the intraindividual variability in sleep onset and wake-up times. Therefore, we used the same kind of analysis as for mid-sleep. As participants fell asleep before and after midnight, we subtracted 24 from the times before midnight to have the scores centered on midnight.

##### Sleep onset

The intercept-only model showed that intraindividual variability in daily sleep onset (2.21, SD = 1.49) was higher than the interindividual variability (1.91, SD = 1.38). According to the *ICC*, 46.37% of the variance was due to between-participant effects. Our final model that included *workday today* and *workday yesterday* showed a significant main effect for *workday today, F*(1, 99.35) = 25.98, *p* < 0.001. There was no significant effect of *workday yesterday, F*(1, 109.03) = 2.76, *p* = 0.099, and the interaction between the two factors was also not significant, *F*(1, 1002.07) = 0.00, *p* = 0.996. Thus, our results indicate that the participants fell asleep later when it was a free day on the next day. The intraindividual variability of sleep onset (1.90, SD = 1.38) was 0.90 times the size of the interindividual variability (2.12, SD = 1.46).

##### Wake-up time

The intercept-only model indicated that the intraindividual variability in daily wake-up time (1.86, SD = 1.36) was larger than the interindividual variability (1.36, SD = 1.17). The *ICC* indicated that 42.24% of variance could be explained by between-subject effects. We then came up with models that included *workday today* and *workday yesterday*. Our final model revealed a main effect for *workday today, F*(1, 110.03) = 73.90, *p* < 0.001, and *workday yesterday, F*(1, 103.69) = 8.68, *p* = 0.004. We also found a significant interaction between the two factors, *F*(1, 89.41) = 8.01, *p* = 0.006. These results indicate that participants woke up later when they both went to bed and woke up on a free day. However, when participants got up on a workday, it did not matter whether it was a free or workday the day before. The intraindividual variability of the wake-up time (1.35, SD = 1.16) was 1.05 times the size of the interindividual variability (1.29, SD = 1.14).

#### Correspondence Between the Retrospective Assessments of Mid-sleep (MCTQ) and Actigraphy-derived Mid-sleep With Average Daily Mid-sleep Scores Over the Period of 14 Days

Finally, we examined the correspondence of retrospective assessments of mid-sleep and actigraphy-derived mid-sleep with the average daily mid-sleep scores on free and workdays over the period of 2 weeks. To this aim, we used the two recall-based mid-sleep scores on free and workdays obtained with the MCTQ before the experience sampling study (MSF_MCTQ_ and MSW_MCTQ_, respectively), the actigraphy-derived scores of mid-sleep (MSF_ACT_ and MSW_ACT_), and the average scores of mid-sleep on free (MSF_ES_) and workdays (MSW_ES_) that were calculated on the basis of the participants’ reported daily sleep times during the 2 weeks of the experience sampling study.

On free days, the retrospective (MSF_MCTQ_) and the average daily scores of mid-sleep across 2 weeks (MSF_ES_) were correlated at *r* = 0.73 whereas the correlation between the respective scores on workdays (i.e., MSW_MCTQ_ and MSW_ES_) was *r* = 0.79, both correlations significant at *p* < 0.001. The two correlations did not differ significantly from each other, *z* = 1.04, *p* = 0.300.

The actigraphy-derived score for mid-sleep on free days (MSF_ACT_) correlated at *r* = 0.80 with the average daily scores of mid-sleep across 2 weeks (MSF_ES_) whereas the correlation between the respective scores on workdays (i.e., MSW_ACT_ and MSW_ES_) was *r* = 0.97, both correlations significant at *p* < 0.001. The two correlations differed significantly from each other, *z* = −6.74, *p* < 0.001.

#### Discussion

##### Intraindividual Variability of Mid-Sleep Over the Period of 14 Days

The intercept-only model (first model) showed that about half the variance in daily mid-sleep scores across the period of 14 days can be explained by within-person differences and the other half by between-person differences, suggesting that people’s daily mid-sleep scores fluctuate as much within-person from day to day as they do between participants. This is similar to the findings of a 14-day experience sampling study by Buysse and colleagues (2009) that also found that there was substantive intraindividual variability in various quantitative and qualitative sleep measures (e.g., bedtime and wake-up time) assessed with sleep diaries and actigraphy.

However, when we included free and workdays into a mixed model, we found that the intraindividual variability in daily mid-sleep was 0.71 times the size of the interindividual variability, so that daily mid-sleep scores differed less within than between participants. This might be due to the differences in the amount of free and workdays participants had in a week. It supports the findings of previous studies that have shown that the interindividual variability in circadian rhythms is greater than the intraindividual variability, both in humans and animals (see, for example, Sharma, 1996; Selmaoui and Touitou, 2003; Refinetti and Piccione, 2005; Romeijn and Van Someren, 2011; Wassmer and Refinetti, 2019). Differently from Selmaoui and Touitou (2003), we tested participants in their natural environments and not in the laboratory which adds to the validity and generalizability of the findings as participants were able to follow their normal work/university and sleep routines during the study period.

Participants’ mid-sleep times were affected by free and workdays so that participants had different sleep routines on free days compared with workdays (i.e., that they systematically went to bed and got up later on free days than on workdays), and yet, they had similar mid-sleep scores on free days (i.e., that on all free days they went to bed and got up around the same time) and on workdays (i.e., that on all workdays they went to bed and got up around the same time) during the study period. The intraindividual variability of sleep onset was 0.90 times the size of the interindividual variability which is similar to the proportion of inter- and intraindividual variability in mid-sleep. However, participants’ wake-up times differed more within than between participants which might be due to the fact that wake-up times on workdays are largely predetermined by social and work/university demands. This aligns with previous results that showed that sleep onset is dependent on chronotype on workdays whereas wake-up time is not (Roenneberg et al., 2003). Thus, the variability of mid-sleep cannot fully be explained by the variability of its composing factors. Please see Supplemental Material S3 for a more detailed discussion about the implications of the results for the MCTQ.

##### Comparison of Retrospective, Actigraphy-Derived, and Average Daily Assessments of Mid-Sleep

The correlations between the retrospective MSF_MCTQ_ and the MSW_MCTQ_ scores with their corresponding average daily mid-sleep scores over the period of 2 weeks were strong and significant (*r*s = 0.73 and 0.79, respectively) and this indicates that the MCTQ (Roenneberg et al., 2003) is a relatively accurate measure to assess participants’ sleeping patterns. Participants might already think of their average bedtimes when filling out the questionnaire since they are asked to report on their typical sleep behavior over the past 4 weeks. Our findings, however, do not support the results of Santisteban et al. (2018) who reported that participants depict their sleep times more accurately on free days than on workdays. On the contrary, our findings indicated that the correlations between the retrospective scores of mid-sleep and experience-sampling-based average daily assessments of mid-sleep were higher on workdays than on free days. Even though the difference between the two correlations was not significant at *p* < 0.05, it seems reasonable to assume that one might retrospectively assess one’s sleeping patterns during the week better than during free days. During the week, one might have a certain routine at what time to go to bed and get up. However, on free days, one might engage in a variety of different activities that are less predictable.

Kühnle (2006) also reported a high ecological validity of the MCTQ when comparing MSF of the MCTQ with the average MSF score of a 6-week long sleep log in people who exhibit a normal chronotype (i.e., the MSF score corrected for sleep debt was between 2.17 and 7.25), *r* = 0.86 (*p* < 0.001). However, the correlation was much higher within the normal chronotype spectrum than among those with either earlier and later chronotypes (MSF corrected for sleep debt below 2.17 or above 7.25); *r*s = 0.56 and 0.41 (*p*s < 0.001), respectively. Our participants were individuals at the end of their adolescence or early adulthood who typically exhibit later chronotypes (Adan et al., 2012), which was also confirmed by the relatively late daily mid-sleep scores we found in our sample. Thus, the ecological validity of the MCTQ might be dependent on chronotype, that is, the variability in MSF may be higher in earlier and later chronotypes than in normal chronotypes. This might make it harder for earlier and later chronotypes to accurately remember their sleep times and thus harder to fill out the MCTQ.

The actigraphy-derived estimates of MSF and MSW were highly correlated with the average daily estimates of MSF and MSW extracted from the sleep diaries (*r*s = 0.80 and 0.97, respectively). This confirms the assumption that actigraphs and sleep diary derived sleep timings show good correlations (Lockley et al., 1999), indicating that participants can estimate quite well at what time they fall asleep and wake up. The estimations seem to be better on work than on free days as participants seem to better remember the sleep times on workdays.

## Study 2: Longer-Term Temporal Stability of Chronotype Across the Life Span

### Method

#### Participants

The participants for Study 2 were a subsample of the Estonian Biobank cohort (currently more than 200,000 participants), which is a large-scale population-based sample of the Estonian adults (Leitsalu et al., 2014). A part of the Estonian Biobank cohort has been followed up longitudinally and, in this study, we use a subsample of the cohort who have completed the MCTQ (Roenneberg et al., 2003) twice. Recruitment and data collection were assisted by a unique network of data collectors, that is, General Practitioners and other medical personnel in private practices and hospitals, but also recruitment offices at the Estonian Genome Center. Participants gave their informed consent which can be found at https://www.geenivaramu.ee/en/access-biobank. Doctors conducted a standardized health examination of each participant. Participants gave blood samples and filled in and completed a Computer Assisted Personal Interview (CAPI) on health-related topics and various clinical diagnoses described in the World Health Organization (WHO) International Classification of Diseases–10th revision (ICD-10) (Leitsalu et al., 2014).

Supplemental Figure S4.1 depicts a flowchart of how participants from the Estonian Biobank were selected. Overall, 1111 participants completed the MCTQ twice over the period of 1 to 9 years. The first time they filled it in was between 2007 and 2010, while the second time was between 2009 and 2016. However, we had to exclude participants either at the first (T1) or at the second (T2) point of measurement due to (a) an average sleep duration of shorter than 4 h, (b) taking medications that influence sleep (categorized with the WHO’s ATC/DDD Index), (c) doing shift work, or (d) missing data. We also excluded 10 participants who had completed the MCTQ for the second time more than 5 years later (i.e., five participants filled in the questionnaires 6 years apart, two 7 years apart, two 8 years apart, and one 9 years apart). It is a well-known fact that stability and consistency generally decline with longer retest intervals, but we did not have enough participants to test this effect in a more systematic way.

The final sample consisted of 681 participants, 344 (50.51%) of them were female. Their mean age at T1 was 47.73 years (SD = 15.89), ranging from 18 to 87 years. At T1, 69 (10.12%) persons had basic education, 363 (53.30%) completed secondary education/secondary vocational education, and 249 (36.56%) completed higher education.

Fifteen participants (2.20%) completed the MCTQ (Roenneberg et al., 2003) for the second time in the same year (ranging from 1 to 11 months apart), 48 (7.05%) completed the questionnaires 1 year apart, 220 (32.31%) 2 years apart, 293 (43.02%) 3 years apart, 54 (7.93%) 4 years apart, and 51 (7.49%) 5 years apart. On average, the time between two measurements was 2.70 years (SD = 1.05), ranging from 0 (40 days) to 5 years. Due to the small number of participants who filled out the MCTQ in the same year, we combined those with the group that completed the MCTQ 1 year apart. We performed a one-way analysis of variance (ANOVA) to test whether the groups with different retest intervals differed in terms of age. The results revealed that the five groups did not significantly differ in age either at T1, *F*(4, 676) = 0.13, *p* = 0.971, or at T2, *F*(4, 676) = 0.77, *p* = 0.545. We also performed a chi-square test of independence to compare the frequency of gender and educational level across the six groups. While the groups did not differ in terms of the highest level of educational attainment, χ^2^(8, *N* = 681) = 2.53, *p* = 0.961, the gender distribution was not equal across the groups, χ^2^(4, *N* = 681) = 31.73, *p* < 0.001, so that there were far fewer women than expected in the group of participants who completed the MCTQ 2 years apart and far more women than expected in the group who were retested 5 years later. Table 1 describes the five groups according to their age, gender, and educational attainment at both time points.

**Table 1.** Sociodemographics and mean scores of mid-sleep on free days and workdays across the five groups who completed the MCTQ twice either 0-1, 2, 3, 4, or 5 years apart (Study 2).

Year	0-1	2	3	4	5	Total
*n*	63	220	293	54	51	681
Age at T1 (SD)	47.16(16.81)	47.68(15.68)	47.90(16.25)	48.67(16.25)	46.69(14.89)	47.73(15.89)
Age at T2 (SD)	48.56(16.86)	50.33(15.71)	51.34(16.31)	52.98(15.00)	52.14(14.90)	50.95(15.96)
Gender						
Female (%)	35(55.56%)	90(40.91%)	143(48.81%)	35(64.81%)	41(80.39%)	344(50.51%)
Education at T1						
Basic (%)	6(9.52%)	25(11.36%)	28(9.56%)	6(11.11%)	4(7.84%)	69(10.12%)
Secondary/vocational (%)	30(47.62%)	114(51.82%)	160(54.61%)	29(53.70%)	30(58.82%)	363(53.30%)
Higher (%)	27(42.86%)	81(36.82%)	105(35.84%)	19(35.19%)	17(33.33%)	249(36.56%)
Mid-sleep scores						
MSF at T1 (SD)	3.83(1.12)	3.81(1.26)	3.76(1.18)	3.81(1.06)	3.77(1.16)	3.79(1.19)
MSF at T2 (SD)	3.62(1.23)	3.82(1.22)	3.68(1.20)	3.76(1.02)	3.58(1.06)	3.71(1.18)
MSW at T1 (SD)	2.91(0.83)	2.90(0.85)	2.92(0.81)	2.94(0.77)	2.82(0.93)	2.91(0.83)
MSW at T2 (SD)	2.80(0.78)	2.98(0.98)	2.92(0.87)	2.86(0.73)	2.80(0.90)	2.92(0.89)

Abbreviations: MCTQ = Munich Chronotype Questionnaire; Year = the difference between the first (T1) and the second (T2) completion of the MCTQ in years; MSF = mid-sleep score on free days; MSW = mid-sleep score on workdays; secondary/vocational = secondary education and secondary vocational education. All percentages are within the specific group (year difference when filling out the questionnaires).

#### Measures

##### MCTQ

The Estonian version of the MCTQ by Roenneberg and his colleagues (2003) was used. It is a 17-item retrospective questionnaire that assesses chronotype. Similar to Study 1, the mid-sleep scores on free (MSF) and work (MSW) days were extracted from the questionnaire. For the sake of consistency with Study 1, we did not correct mid-sleep on free days (MSF) for sleep debt on workdays (MSF_sc_) in Study 2. However, as suggested by an anonymous reviewer, we also repeated all the analyses using MSF_sc_ and found similar trends. The results of these analyses are reported in Supplemental Material S5, Tables S5.1 and S5.2 and Figures S5.1 to S5.4.

#### Analyses

We used IBM SPSS Statistics 24 for statistical analyses. For each participant, we computed Asendorpf’s (1992) coefficient for individual stability both for MSF and MSW to obtain a measure of intraindividual change in rank-order stability over time (cf. Terracciano et al., 2010). This score is calculated as such:

$$i_{12}=1-\frac{{\left(z_{1}-z_{2}\right)}^{2}}{2},$$

where $z_{1}$ and $z_{2}$ are the *z*-transformed scores at T1 and T2. The higher the score is, the more stable are the scores between the two measurement points. A negative score indicates that the scores are less stable. The population mean matches the correlation $r_{12}$ between the two assessments.

Since the coefficients of individual stability were strongly skewed to the left, we transformed the scores as proposed by Asendorpf (1992):

$$Ti_{12}=\{\frac{\frac{1}{2}\ln\left[\frac{1.001+i_{12}}{1.001-i_{12}}\right]for\;0\;\leq i_{12}\leq 1}{\ln\left[\frac{1}{1-i_{12}}\right]for\;i_{12}<0}.$$

We plotted the *t*-transformed scores for MSF and MSW with age and fitted a curve that matched the data best (polynomial curve of two degrees). We divided our participants into age groups to identify how the stability of mid-sleep changes with age. To inform our analyses, we ran a series of hierarchical linear regression models to test whether the *t*-transformed coefficients of individual stability in MSF and MSW were influenced by the age of participants at T1, the quadratic term of age at T1, as well as the time difference between T1 and T2.

### Results

#### Descriptive Statistics

Across all participants, the average mid-sleep score on free days (MSF) was 3.78 (SD = 1.18) at T1 and 3.72 (SD = 1.18) at T2. The scores did not significantly differ from each other *t*(678) = 1.71, *p* = 0.087. The average mid-sleep scores on workdays (MSW) also did not differ between T1 (M = 2.91; SD = 0.83) and T2 (M = 2.92, SD = 0.89), *t*(678) = −0.33, *p* = 0.740. Table 1 gives an overview about these scores according to the year difference between filling out the questionnaires. The correlations between MSF and MSW were *rs* = 0.70 and 0.69 at T1 and T2, respectively (both significant at *p* < 0.001).

#### Test-retest Reliabilities of Mid-sleep Scores for the Groups With Different Retest Intervals

The test-retest correlations for MSF and MSW for the full sample were *r* = 0.66 and *r* = 0.58, respectively (both significant at *p* < 0.001). The test-retest correlations for MSF and MSW for groups with different retest intervals ranging from 0-1 to 5 years are shown in Supplemental Figure S6.1. Broadly speaking, the retest correlations of MSF and MSW were very similar across the groups with different retest intervals, and varied between 0.63 (tested 2 years apart) and 0.70 (tested 3 years apart) for MSF and between 0.51 (tested 1 year apart) to 0.65 (tested 5 years apart) for MSW, respectively. The retest stability of MSF was consistently higher (median retest correlation = 0.65) than the stability of MSW (median retest correlation = 0.54) across all five groups with different retest intervals.

#### Individual and Group-level Stability of Mid-sleep Across the Life Span

Finally, we were interested in finding out if and to what extent the individual stability coefficients for MSF and MSW depend on age. Supplemental Figures S7.1 and S7.2 depict age at the first time of assessment (T1) on the *x*-axis and the Asendorpf’s (1992) *t*-transformed coefficients of individual stability of MSF and MSW on the *y*-axis. A *t*-transformed coefficient of individual stability of 3.8 corresponds to an individual stability coefficient of 1 and a *t*-transformed coefficient of 2.6 to a coefficient of 0.99. As can be seen from Supplemental Figure S7.1, the individual stability of MSF increases from young adulthood to early 50s and then starts to decline again from mid-50s onward. When we fitted a quadratic model on the data (equation of *y* = −0.001*x*² + 0.074*x* – 0.240), it accounted for 3.53% of the variance in MSF individual stability coefficients compared with the linear model, which only accounted for 1.63%. As for MSW, the individual stability coefficients increase from young adulthood until mid-40s and then decrease from late 40s onward. When we fitted a quadratic model on the data, it accounted for 2.40% of the variance in MSW individual stability coefficients compared with the linear model, which only accounted for 0.27%, with an equation of *y* = −0.001*x*² + 0.085*x* – 0.300. In any case, the individual stability of mid-sleep both on free and workdays seems to reach its peak in middle age, namely in 40s and 50s.

**Figure 2.**
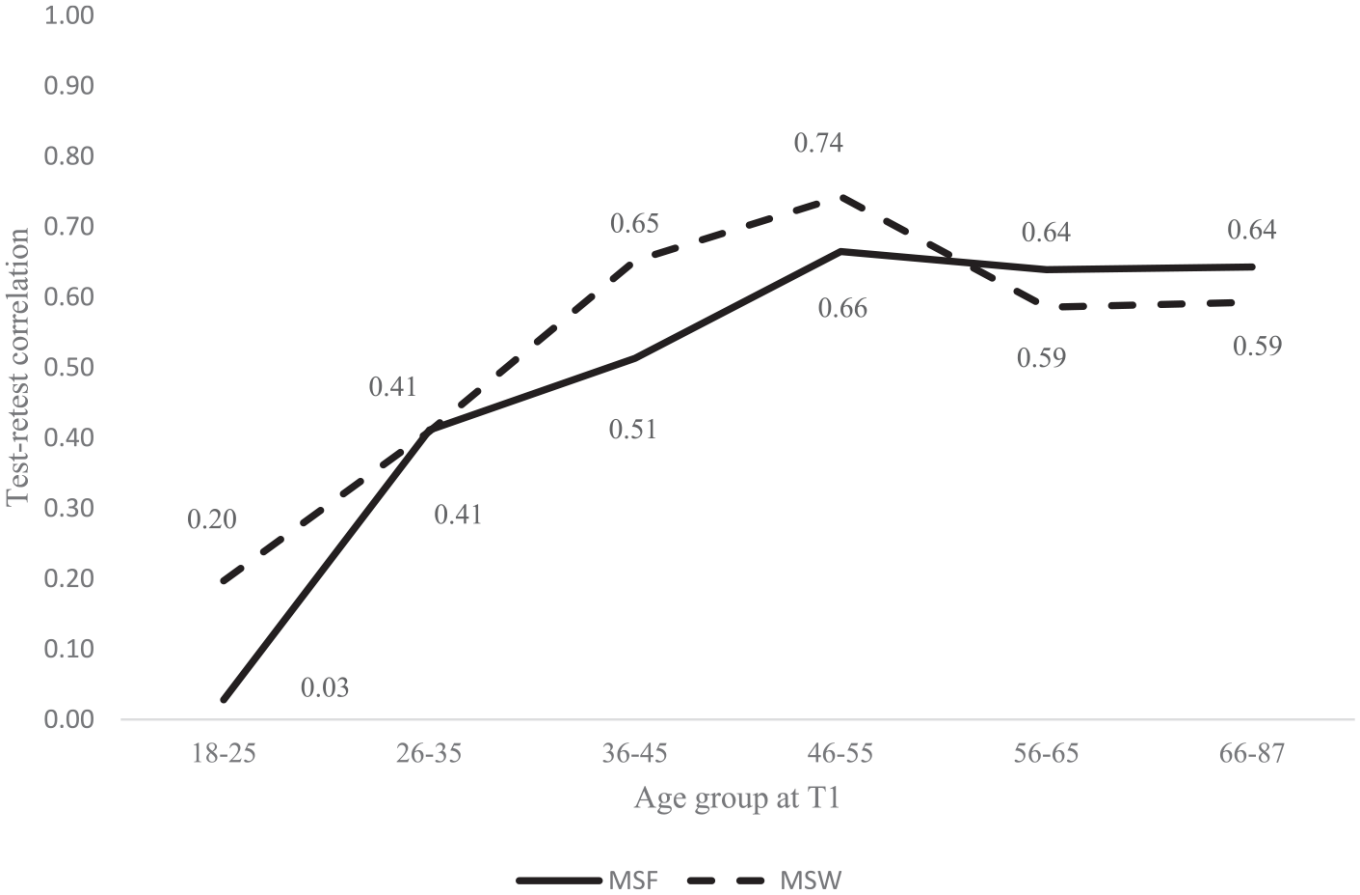
Test-retest correlations of MSF and MSW by age at T1 (Study 2). Abbreviations: MSF = mid-sleep on free days; MSW = mid-sleep on workdays.

To further elaborate on how the rank-order stability of MSF and MSW is influenced by age, we divided participants into six age categories at T1: 18 to 25 (*n* = 69), 26 to 35 (*n* = 114), 36 to 45 (*n* = 134), 46 to 55 (*n* = 121), 56 to 65 (*n* = 124), and 66 to 87 (*n* = 119). We then calculated test-retest correlations for MSF and MSW for each group. Figure 2 depicts these test-retest correlations by age group. The rank-order stability of MSF seems to reach a plateau when participants are in late 40 to early 50 years of age (*r* = 0.66, *p* < 0.001) whereas the rank-order stability of MSW also reaches its peak when participants are 46 to 55 years old (*r* = 0.74, *p* < 0.001) and then decreases again in older participants.

Since stability of psychological traits tends to decline with longer retest intervals (Roberts and DelVecchio, 2000; Terracciano et al., 2006) and because the retest interval varied across the participants of our study, we ran a series of hierarchical regression analyses where we predicted the individual stability coefficients (MSF and MSW in separate models) from participant’s age and the square of age at T1 (to account for both linear and non-linear relationships) when also controlling for retest interval. The results of the hierarchical multiple regression analyses for the individual stability of MSF and MSW are shown in Supplemental Material (Table S8.1 and S8.2, respectively). In both models, age and age square had a significant effect on the stability of mid-sleep at *p* < 0.001. Time difference in the retest interval was a significant predictor of the stability of MSF at *p* = 0.045 but not of the stability of MSW (*p* = 0.477).

#### Discussion

Previous cross-sectional and a few longitudinal studies have shown that chronotype changes across the life span (Koskenvuo et al., 2007; Adan et al., 2012; Broms et al., 2014). We wanted to find out whether there was any systematic variation in the longer-term stability of chronotype across life span. Our results indicate that the rank-order stability of mid-sleep on both free and workdays varies with age and is the highest when participants are in their late 40s to early 50s.

In their most recent critical review of their work, Roenneberg et al. (2019) argued that chronotype should rather be seen as a state and not a trait since zeitgeber signals people are exposed to vary in strength and timing. This might indicate that the life circumstances of younger and older people may vary more than those of middle-aged participants. During adulthood, humans experience a variety of major life events which in turn might have an impact on their bedtimes. MSF seems to change the most when participants are at the age of starting something new, for example, a job (de Souza et al., 2014), living together with a partner (Hida et al., 2012), or starting a family (Leonhard and Randler, 2009). Therefore, it is not as surprising that the stability of MSF reached a plateau in the age group of 46 to 55 years of age who most likely had already experienced such life events. MSW also reached its peak of stability in the same age group (i.e., 46-55 years) but decreased again in older age groups, meaning that people’s sleep habits on workdays seem to become less stable when they reach the retirement age (Hagen et al., 2016) and when their daily routines are no longer determined by work and school hours.

Due to the nature of our study, participants filled out the questionnaire for the second time at different years apart from each other. The results of the hierarchical regression analyses showed that age and its square were more important than the year difference when predicting the stability of MSF and that the year difference in filling out the questionnaires did not matter when predicting the stability of MSW. A possible reason for this could be that the time difference between filling out the questionnaires was quite small, ranging from 0 to 5 years.

Overall, both MSF and MSW showed strong test-retest correlations when participants filled out the MCTQ up to 5 years apart from each other. The retest stability of MSF was higher than the retest stability of MSW at both time points, which shows that one’s bedtimes on free days are more stable than those on workdays. Future studies should establish how longer time intervals between filling out the questionnaires will affect the stability of both MSF and MSW.

## General Discussion

Even though chronotype has been a topic of extensive research over the past decades, most studies have used preferential (e.g., The Morningness-eveningness Questionnaire; Horne and Östberg, 1976) or retrospective recall-based measures of chronotype (e.g., MCTQ; Roenneberg et al., 2003), which typically do not examine the daily intraindividual variability in chronotype. Research in different fields of psychology suggests that global retrospective measures, especially summary measures that ask participants to report on their typical behavior over several past weeks or months, are often biased because people use mental heuristics to recall information (Shiffman et al., 2008). Study 1 aimed to fill this gap in literature and contribute to a better understanding of intraindividual variability of chronotype over a period of 2 weeks, as well as to examine correspondence between recall-based estimates of chronotype (i.e., MCTQ) and actigraphy-derived estimates of mid-sleep with average real-time estimates of MSF and MSW. Furthermore, only few studies have investigated the temporal stability of chronotype; the majority of those studies evaluated the test-retest reliability of chronotype questionnaires during relatively short periods of time while not bearing in mind how age might affect the temporal stability of chronotype (see, for example, Smith et al., 1989; Kühnle, 2006; Di Milia et al., 2013). Thus, Study 2 examined the stability of mid-sleep over longer periods of time while also considering the effect of age.

When the daily variability in mid-sleep was examined across the study period of 2 weeks (Study 1), we found that the intraindividual variability was about equal to the interindividual variability in daily mid-sleep scores (*ICC* = 50.46%), meaning that there was as much variability between participants’ daily mid-sleep scores as in within each participant. However, when the effect of free versus workday was considered, people’s mid-sleep scores fluctuated more across than within participants. Our findings also speak for the relatively high levels of intraindividual consistency in chronotype, meaning that even though people have different mid-sleep points on work and free days, they tend to have a routine of going to bed and getting up on workdays and another routine on free days. We also found that waking up on a free day has the biggest influence on one’s mid-sleep—not surprisingly, people wake up later on free days than on workdays—but interestingly, going to bed on a free day also delays one’s mid-sleep, meaning that people go to bed and wake up the latest when both the day they go to bed and the day they wake up are free days.

The recall-based retrospective mid-sleep scores on free (MSF) and work (MSW) days extracted from the MCTQ (Roenneberg et al., 2003) correlated highly with the respective average mid-sleep scores from the experience sampling study (*r*s = 0.73-0.79). This is consistent with previous research (Kühnle, 2006; Kantermann et al., 2015; Santisteban et al., 2018) and speaks for high ecological validity of the MCTQ. It seems though that our participants were slightly more accurate in retrospectively estimating their sleep times on workdays than on free days which could be explained by the fact that there are more restrictions and less flexibility in sleep times due to university-related responsibilities (e.g., classes, seminars) on weekdays compared with free days (cf. Paine and Gander, 2016), and thus, sleep times can be more accurately recalled. However, it should be noted that the sleeping times assessed in the MCTQ asked about the 4 weeks before the start of the experience study and therefore did not overlap with the sleeping times extracted from the experience sampling study. This means that the bedtimes extracted from the MCTQ could have also differed from the average bedtimes during the course of the experience sampling study. As we found high correlations between the average daily self-reported mid-sleep scores and the average mid-sleep scores assessed with actigraphy during the same period of 14 days, we can be quite certain that participants can estimate well at what times they fall asleep and wake up. However, the correlations might be this high because we anchored the actigraphy-derived sleep times on the sleep times extracted from the sleep diaries.

Study 2 contributed to important insights into the change and stability of chronotype over a longer period of time and across different stages of life span. We found relatively high retest correlations for MSF and MSW when examining the retest stability of mid-sleep during the periods of 0-1 to 5 years. The median retest correlations of MSF and MSW at T1 with T2 across different time periods were 0.65 and 0.54, respectively, which are comparable (if slightly lower) with the retest stability coefficients of the Big Five personality traits assessed in middle adulthood with a testing interval of 3 to 10 years (Hampson and Goldberg, 2006; Terracciano et al., 2010). Our estimates were, however, a bit lower than those reported in previous studies when participants’ chronotype was tested twice during 1 to 24 months using mostly preferential questionnaires of chronotype or morningness and eveningness (see, for example, Smith et al., 1989; Greenwood, 1994; Caci et al., 2000; Griefahn et al., 2001; Kühnle, 2006; Wood et al., 2009; Di Milia et al., 2013). The lower retest stability indicators in our study could be due to a longer time span between the two measurements since it is known that the stability of psychological traits declines with longer test-retest intervals (Roberts and DelVecchio, 2000; Terracciano et al., 2006). As our testing interval varied from 0-1 to 5 years, we also conducted hierarchical linear regression analyses to examine the effects of age and the year difference on the stability of mid-sleep. The results showed that age was more important than the year difference between two measurements in predicting the stability of MSF. The stability of MSW was only affected by age and not the year difference between the measurements.

Interestingly, across the whole sample, the retest stability of MSF (0.66) was greater than on workdays (0.58) over the periods of up to 5 years. During a longer time interval, several life circumstances might change due to changing opportunities and constraints characteristic of different stages in life (Heckhausen et al., 2010). These could have affected one’s MSW, for example, having children and their entry into school, getting a promotion, or retiring.

One of the novel aspects of Study 2 was to examine the retest stability of chronotype across different stages of life span. Even though MSF and MSW remained relatively stable over a period of up to 5 years, the retest stability varied greatly in different age groups: the retest stability coefficients both for MSF and MSW were the lowest when participants were in late teens and early 20s and the highest when participants were in their late 40s to early 50s. Even though mid-sleep fluctuates little within young adults over a period of 2 weeks (Study 1), the temporal stability coefficients of mid-sleep are very low when participants are tested twice over much longer periods of time (Study 2). Interestingly though, the stability of MSF reaches a plateau and levels off when participants are in their late 40s to early 50s whereas the stability of MSW decreases again when people reach the retirement age. This seem to suggest that the differences in the stability of mid-sleep across the life span are likely not solely due to biological age effects but also to social life-cycle effects (e.g., finishing school, finding a job, getting married, settling down, retiring) that are intertwined with the biological process of aging (Glenn, 2003). These findings are confirmed by individual-level stability analyses (see Supplemental Figures 7.1 and 7.2), which provided further evidence in support of the view that mid-sleep stability changes over the life span. Overall, both group and individual-level analyses clearly indicate that when examining the stability of chronotype or mid-sleep, the effect of age (either biological or social in nature) strongly needs to be considered.

### Limitations and Future Research

Our approach was not without limitations though. First, the participants from both studies differed in age. The participants from Study 1 were university students whereas the participants from Study 2 were part of a large-scale sample of Estonian adults ranging from 18 to 87 years in age at T1. Therefore, the results of Study 1 might not be applicable to older participants whereas we did consider the effect of age in Study 2.

In Study 1, we were able to show that intraindividual variability in mid-sleep is a lot smaller than interindividual variability when the type-of-day variable was controlled for. This can be partly explained by the difference in the amount of free and workdays our participants reported. Future research could use experience sampling methodology of mid-sleep using a representative population over a longer period of time to generalize our findings of intraindividual stability of chronotype. It would also be interesting to explore whether early and late chronotypes show a different intraindividual variability in mid-sleep.

When examining the temporal stability of mid-sleep across the life span in Study 2, we compared the stability of mid-sleep in different age groups that consisted of different participants. Thus, future studies need to confirm our findings by applying a longitudinal approach that would allow to examine the intraindividual change of the stability of mid-sleep in the same individuals across the life span (cf. Terracciano et al., 2010). Furthermore, long-term longitudinal studies will be necessary to explore how life circumstances and other possible factors influence one’s mid-sleep.

In sum, our studies have given important insights on the intraindividual variability and temporal stability of mid-sleep. Using experience sampling and longitudinal methodologies, we were able to complement the weaknesses of cross-sectional studies. Our results show that mid-sleep varies less within than between participants when the effect of free and workdays is controlled for and that the stability of mid-sleep of both MSF and MSW is largely dependent on age. However, future research is needed to investigate how intraindividual variability of mid-sleep is dependent on chronotype and how the temporal stability of mid-sleep systematically changes with age.

## Supplemental Material

sj-pdf-1-jbr-10.1177_0748730420974842 – Supplemental material for Intraindividual Variability and Temporal Stability of Mid-Sleep on Free and Workdays Click here for additional data file. Supplemental material, sj-pdf-1-jbr-10.1177_0748730420974842 for Intraindividual Variability and Temporal Stability of Mid-Sleep on Free and Workdays by Anita Lenneis, Ahuti Das-Friebel, Henrik Singmann, Maris Teder-Laving, Sakari Lemola, Dieter Wolke, Nicole K. Y. Tang, Adrian von Mühlenen, Jüri Allik and Anu Realo in Journal of Biological Rhythms
