# Supplementary material for: Intraindividual Variability and Temporal Stability of Mid-Sleep on Free and Workdays
Source: J Biol Rhythms. 2020 Dec 22;36(2):169–84. doi: 10.1177/0748730420974842 (PMC8056704; doi:10.1177/0748730420974842)
Supplement: sj-pdf-1-jbr-10.1177_0748730420974842 – Supplemental material for Intraindividual Variability and Temporal Stability of Mid-Sleep on Free and Workdays [file sj-pdf-1-jbr-10.1177_0748730420974842.pdf]

Supplemental Materials

Supplemental Material S1

Table S1.1

Review of Studies Investigating Test-Retest Reliabilities of Preferential Morningess-Eveningness and Sleep Time Based Assessments of Chronotype

Study	Measurement	*N*	Time duration	*r*	*p*	Age span in years	Mean age in years (*SD*)
Preferential Questionnaires							
Pündük et al. (2005)	Morningness-Eveningness Questionnaire (MEQ; Horne and Östberg, 1976)	618	15-20 days	.84	< .001	18 to 57	23.00 (5.40)
Lee et al. (2014)	MEQ	21	4 weeks	.90	<.001	20-39*	N/A
Griefahn et al. (2001)	MEQ	43	7 to 12 weeks	.97	<.001	18-68*	23.4 (7.1)*
Neubauer (1992)	MEQ	48	2 months	.89	N/A	18-47*	N/A
Greenwood (1994)	Composite Scale of Morningness (CSM; Smith et al., 1989)	36	3 months	.91	<.001	18-52*	21.3*
Larsen (1985)	MEQ	74	3 months	.88	N/A	N/A (University students)	N/A
Greenwood (1994)	CSM	35	9 months	.82	<.001	18-52*	21.3*
Caci et al. (2000)	CSM	60 (49 females, 11 males)	13 months	.93 (females) .86 (males)	<.001	18-40	22.51 (4.37)

Kantermann and Eastman (2018)	MEQ	18	9 months to 3 years	.85	<.001	21-44	N/A
Wood et al. (2009)	CSM	52	2 years	.72	<.001	N/A (adults, offspring between 2 and 18 years)	N/A
Reports of Sleep Times							
Reis et al. (2020)	Munich Chronotype Questionnaire (MCTQ; Roenneberg et al., 2003)	41	2-6 weeks	rho = .83 (MSW), rho = .83 (MSF), rho = .91 (MSF_{sc})		18-64	44.12 (14.54)
Kühnle (2006)	MCTQ	15	up to 3 weeks	.81	< .001	N/A	N/A
Suh et al. (2018)	MCTQ	78 (only females)	3 months	.73 (MSW), .72 (MSF), .72 (MSF_{sc})	< .001	20-27	21.83 (1.67)
Kühnle (2006)	MCTQ	96	Aug-Sept to January (5-6 months)	.88	< .001	N/A	N/A
McMahon et al. (2018)	MCTQ	390	6, 12, 18, and 24 months	test-retest correlations not reported; MSF_{sc} declined over time; shift to earlier chronotype only observed in late chronotype group		21-35	27.6 (3.8)

Kantermann and Eastman (2018)	MCTQ	18	9 months to 3 years	.78	< .001	21-44	N/A
Urner et al. (2009)	Actigraphic MSW and MSF	23	5 years	.58 (MSW), .55 (MSF)	<.05	17-19 at T1	18.4 (0.9) at T1; 23.4 (0.9) at T2
Druiven et al. (2020)	MCTQ	1,417	7 years	.53	<.001	18-65	N/A

Note. *r* = test-retest correlation; *N* = sample size; MSW = mid-sleep on workdays; MSF = mid-sleep on free days; MSF_{sc} = mid-sleep on free days corrected for sleep debt; * = only a subsample filled out the questionnaires twice, N/A = not available; but only the age of the whole sample was reported; T1 = first time of assessment; T2 = second time of assessment

Supplemental Material S2

We excluded 21 instances due to several reasons in the following order: Six instances because participants had indicated the same wake up and going to bed times, one because they went to bed before trying to fall asleep, one because they needed more than 5 h to fall asleep, six because their sleep duration was less than or equal to 1 h, one because their sleep duration was more than 15 h, three because their mid-sleep score was more than 15, and finally three because there was no information available on whether it was a work or free day.

Supplemental Material S3

If we also consider the strong correlations between the mid-sleep scores on free and workdays (both at the level of retrospective [i.e., MCTQ] and daily average measurements, *rs* = 0.84 and 0.87, respectively), our findings indicate that people seem to have a general disposition which makes them go to bed either earlier or later, regardless of whether it is a work- or free day. Our final model also showed that participants had a later mid-sleep score when they woke up on a free day and went to bed on a free day. Seizing the opportunity to sleep in on a free day might lead to going to bed later which in turn might also influence one’s wake-up time as suggested by the models examining sleep onset and wake-up time. Therefore, when asking about one’s MSF, a free day should be defined as a day when one can go to bed and get up on a free day, which would be a Sunday in a typical European workweek. In the MCTQ (Roenneberg et al., 2003), for example, participants are asked to differentiate between free and workdays when talking about their sleeping patterns but it is not properly defined what a free day actually means.

Supplemental Material S4

Figure S4.1. *Flowchart of the Study 2 sample selection process (Estonian Biobank).*

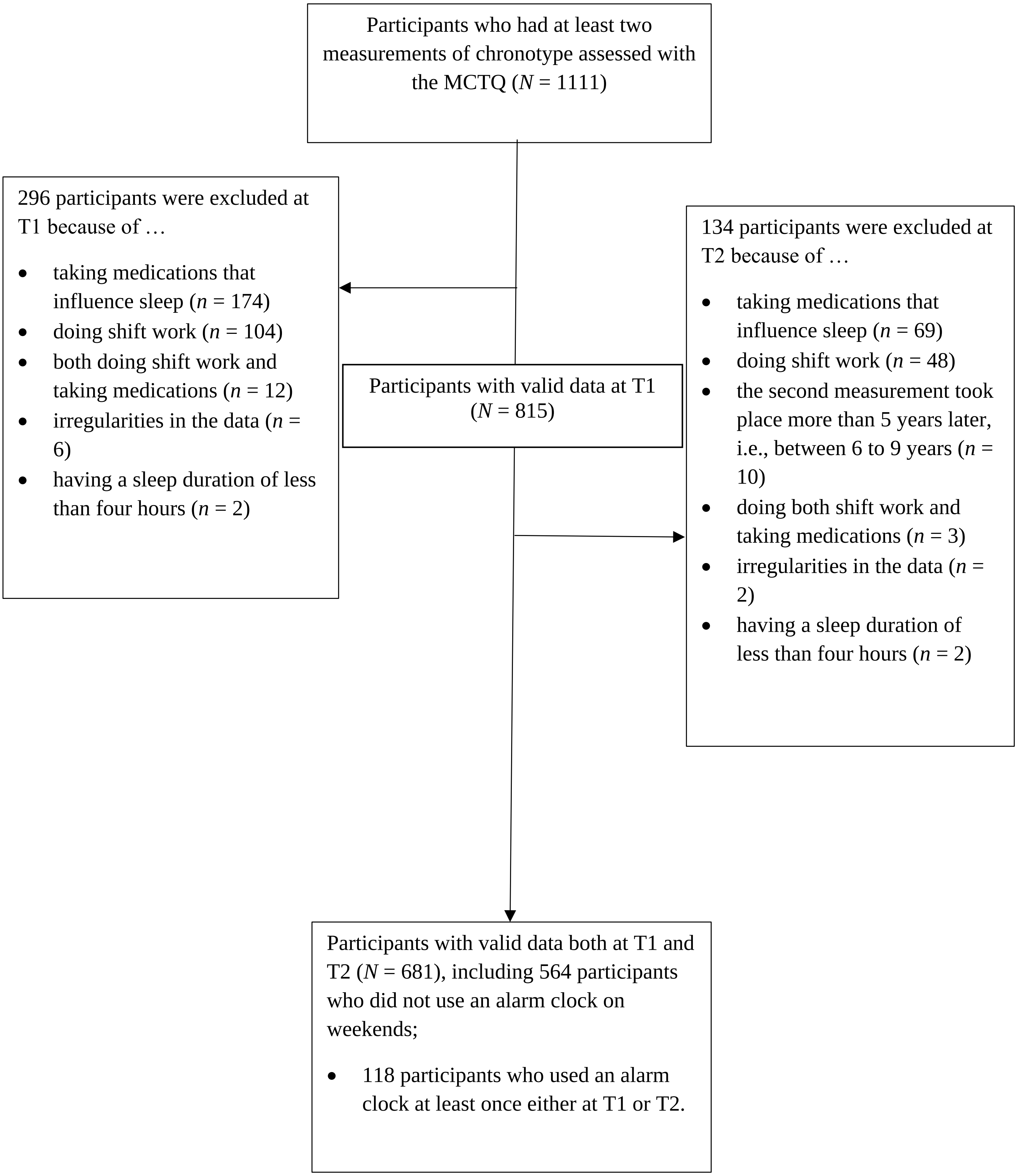

Supplemental Material 5: Temporal Stability of Mid-Sleep Using Mid-Sleep on Free Days Corrected for Sleep Debt (MSF_{sc}) and Mid-Sleep on Free Days (Study 2)

As chronotype is often operationalized as mid-sleep on free days corrected for sleep debt (MSFsc; Roenneberg et al., 2003; Roenneberg, 2015), we wanted to ensure that our analyses were not influenced by the decision to only investigate mid-sleep on free days (MSF). MSF_{sc} can only be calculated if participants do not use an alarm clock on weekends. If the sleep duration on free days is smaller than or equal the sleep duration on workdays, MSF does not need to be corrected. When the sleep duration on free days is greater than the sleep duration on workdays, it is calculated as such:

$$MSF_{\mathrm{sc}} = MSF - \frac{(SD_{free} - SD_{week})}{2}$$

where SD_{free} is equal sleep duration on free days and SD_{week} is equal sleep duration on workdays.

We had to exclude 118 participants because they had used an alarm clock on free days at either the first or second time of assessment. Therefore, our final sample consisted of 563 participants. As the exclusion of participants might have impacted our results, we re-ran the analyses for both MSF and MSF_{sc}. The mean age of the participants at T1 was 49.57 (SD = 15.51). Around half of the participants identified as female (272; 48.31%). At T1, 58 (10.30%) participants had basic education, 309 (54.88%) had completed secondary education/secondary vocational education, and 196 (34.81%) had higher education.

Results

Descriptive Statistics

Across all participants, the average MSF was 3.71 (SD = 1.18) at T1 and 3.65 (SD = 1.19) at T2. The scores did not significantly differ from each other, $t(562) = 1.44$, $p = .150$. The MSF_{sc} score at T1 was 3.39 (SD = 1.02) and 3.38 (SD = 1.08) at T2, $t(562) = 0.28$, $p = .780$. The correlation between MSF and MSF_{sc} at both T1 and T2 was $r = .95$ ($p < .001$),

MSF and MSF_{sc} differed from each other significantly at both T1, $t(562) = 19.96$, $p < .001$ and T2, $t(562) = 18.12$, $p < .001$.

Test-retest Reliabilities of Mid-Sleep Scores for the Groups with Different Retest Intervals

The test-retest correlations for this sample were $r = .67$ for MSF and $r = .59$ for MSF_{sc}. All correlations significant at $p < .001$. The test-retest correlations for groups with different test intervals ranging from 0-1 to 5 years of MSF and MSF_{sc} are depicted in Figure S5.1. In general terms, the stability of the two variables remained quite high over the course of the years. The overall highest test-retest correlations were found for MSF which ranged from $r = .64$ (2 years) to $r = .74$ (0-1 year) which were slightly higher than the test-retest correlations of MSF_{sc} with *r*s ranging from .57 (2 years) to $r = .64$ (0-1 and 3 years). However, the test-retest correlations for MSF and MSF_{sc} according to the retest interval did not differ from each other significantly at $p < .05$.

Individual and Group-Level Stability of Mid-Sleep Across the Life Span

Next, we examined how the stability coefficients of MSF and MSF_{sc} depend on age. Figure S5.2 and Figure S5.3 depict age at T1 on the x-axis and Asendorpf's (1992) *t*-transformed coefficients of individual stability of MSF (Figure S5.2) and MSF_{sc} (Figure S5.3) on the y-axis. A *t*-transformed coefficient of individual stability of 3.8 corresponds to an individual stability coefficient of 1 and a t-transformed coefficient of 2.6 to a coefficient of 0.99. The individual stability of both MSF and MSF_{sc} increases from young adulthood to early 50s and then starts to decline again from mid-50s onwards. We added a quadratic fit to both models which explained 5.47 (equation: $y = -0.001x^2 + 0.094x - 0.740$) and 5.32 (equation: $y = -0.001x^2 + 0.097x - 0.945$) percent of the variance in individual stability. Adding the quadratic terms to both models explained about three percent more of the variance.

To further elaborate on how the rank-order stability of MSF and MSF_{sc} is influenced by age, we divided participants into six age categories at T1: 18-25 (n = 47), 26-35 (n = 76), 36-45 (n = 110), 46-55 (n = 108), 56 to 65 (n = 112), and 66-87 (n = 110). We then calculated test-retest correlations for MSF and MSF_{sc} for each group. Figure S5.4 illustrates these test-retest correlations by age group. The rank-order stability for all three variables seems to reach its peak when participants are around 46-55-years old (rs ranging from .71 to .74, ps < .001) and then slightly decreases and reaches a plateau until older age. The test-retest correlations of MSF and MSF_{sc} of each age group did not differ from each other significantly.

As in the main study, we again ran a series of hierarchical regression analyses where we predicted individual stability coefficients (MSF and MSF_{sc} in separate models) from participant's age and the square of age at T1 (in order to account for both linear and non-linear relationships) when also controlling for retest interval. The results of the hierarchical multiple regression analysis for the individual stability of MSF and MSF_{sc} are shown in Table S5.1 and Table S5.2. In the models explaining the stability of MSF and $MSFs_{c}$, age and age square had a significant effect on the stability of mid-sleep and the addition of the variables resulted in significant improvements of the models. However, the time difference in the retest interval was not a significant predictor of stability.

Conclusion

We repeated all the analyses using both MSF and MSF_{sc} in order to ensure that our analyses were not impacted by the decision to solely look into MSF and not MSF_{sc}. As MSF_{sc} can only be calculated when participants are not using an alarm clock on weekends, we had to exclude 118 participants who either had used an alarm clock at T1 or T2.

The test-retest correlations of MSF and MSF_{sc} when looking into the effect of age and time interval of filling in the questionnaire show very similar patterns. However, the coefficients seem to be slightly, but not significantly, higher in MSF than MSF_{sc}, indicating

that participants' chronotypes change more when sleep debt is accounted for. This might be because even if sleep times remain the same, work hours or social demands might have changed over time.

Table S5.1

Hierarchical Linear Regression Analysis Predicting Asendorpf's (1992) t-Transformed Coefficient of Individual Stability of Mid-Sleep on Free Days (MSF) From Age at T1, its Quadratic Term, and. the Difference in Years Between T1 and T2

	Model 1				Model 2				Model 3			
	B [CI]	*β*	*t*	*p*	*B [CI]*	*β*	*t*	*p*	*B [CI]*	*β*	*t*	*p*
**Block 1: Age at T1**												
Constant	1.14 [0.82, 1.46]		7.07	<.001	-0.01 [-0.9, 0.88]		-0.02	.986	0.15 [-0.77, 1.08]		0.33	.744
Age	0.01 [0.00, 0.02]	0.12	2.83	.005	0.06 [0.02, 0.10]	0.83	3.12	.002	0.06 [0.02, 0.1]	0.84	3.16	.002
**Block 2: Quadratic age**												
Quadratic term of age					0.00 [0.00, 0.00]	-0.72	-2.70	.007	0.00 [0.00, 0.00]	-0.73	-2.75	.006
**Block 3: Time difference**												
Time difference									-0.07 [-0.16, 0.03]	-0.06	-1.36	.175
Adjusted R^2	.012				.023				.025			
F for Change in R^2	7.99			.005	7.31			.007	1.84			.175
Effect size f^2	0.014				0.027				0.031			

Note. CI = A 95% confidence interval.

Table S5.2

Hierarchical Linear Regression Analysis Predicting Asendorpf's (1992) t-Transformed Coefficient of Individual Stability of Mid-Sleep on Free Days Corrected From Sleep Debt (MSF_{sc}) From Age at T1, its Quadratic Term, and the Difference in Years Between T1 and T2

	Model 1				Model 2				Model 3			
	B [CI]	*β*	*t*	*p*	*B [CI]*	*β*	*t*	*p*	*B [CI]*	*β*	*t*	*p*
**Block 1: Age at T1**												
Constant	0.91 [0.58, 1.24]		5.40	<.001	-0.02	0.00	-2.01	.045	-0.01		-1.98	.048
Age	0.01 [0.01, 0.02]	0.15	3.64	<.001	0.14	1.24	4.75	<.001	0.14	1.24	4.74	<.001
**Block 2: Quadratic age**												
Quadratic term of age					0.00	-1.11	-4.22	<.001	0.00	-1.10	-4.21	<.001
**Block 3: Time difference**												
Time difference									0.11	0.01	0.14	.893
Adjusted R^2	.021				.050				.048			
F for Change in R^2	13.24			<.001	17.83			<.001	0.02			.893
Effect size f^2				0.024		0.056				0.056		

Note. CI = A 95% confidence interval.

Figure S5.1. *Test-retest correlations of MSF (mid-sleep on free days) and MSF_{sc} (mid-sleep on free days corrected for sleep debt) according to the time difference between the two measurements.*

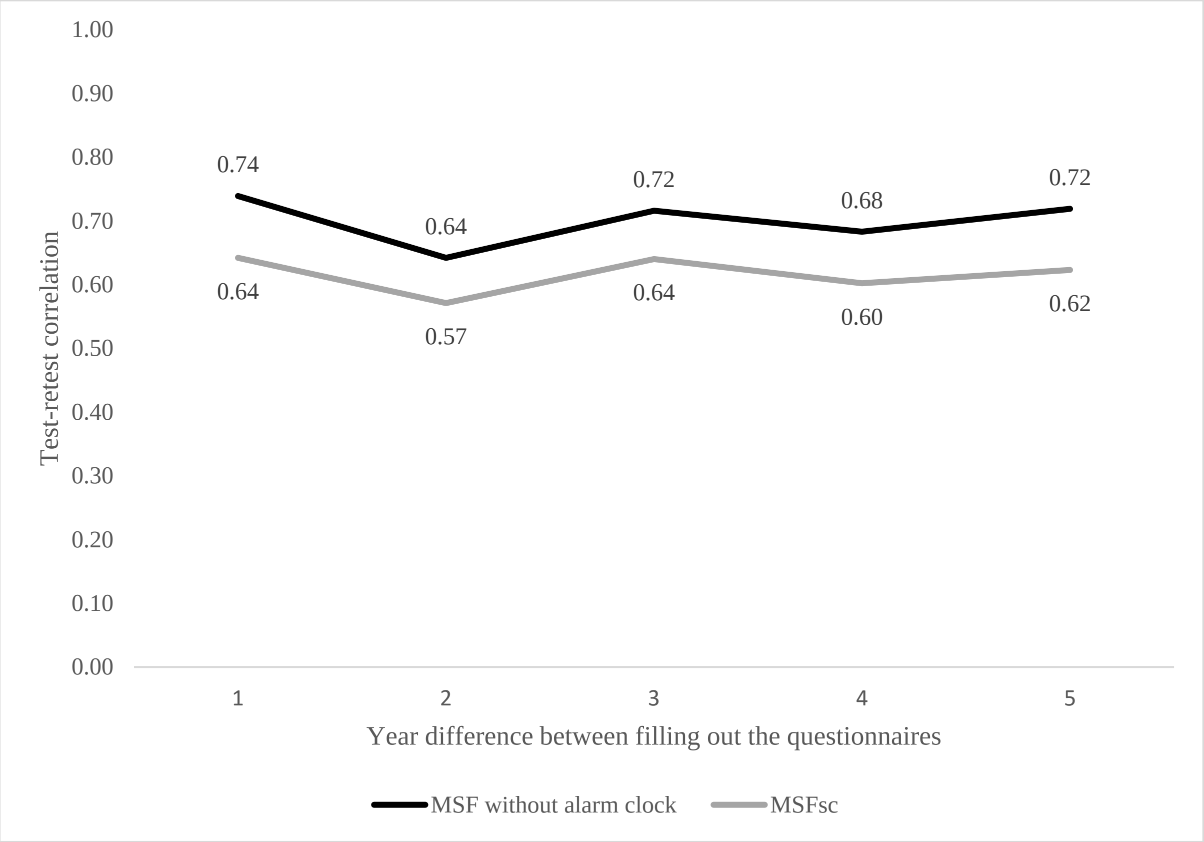

Figure S5.2. *Scatterplot depicting age at T1 on the x-axis and the t-transformed Asendorpf's coefficient of individual stability of MSF (mid-sleep on free days) on the y-axis.*

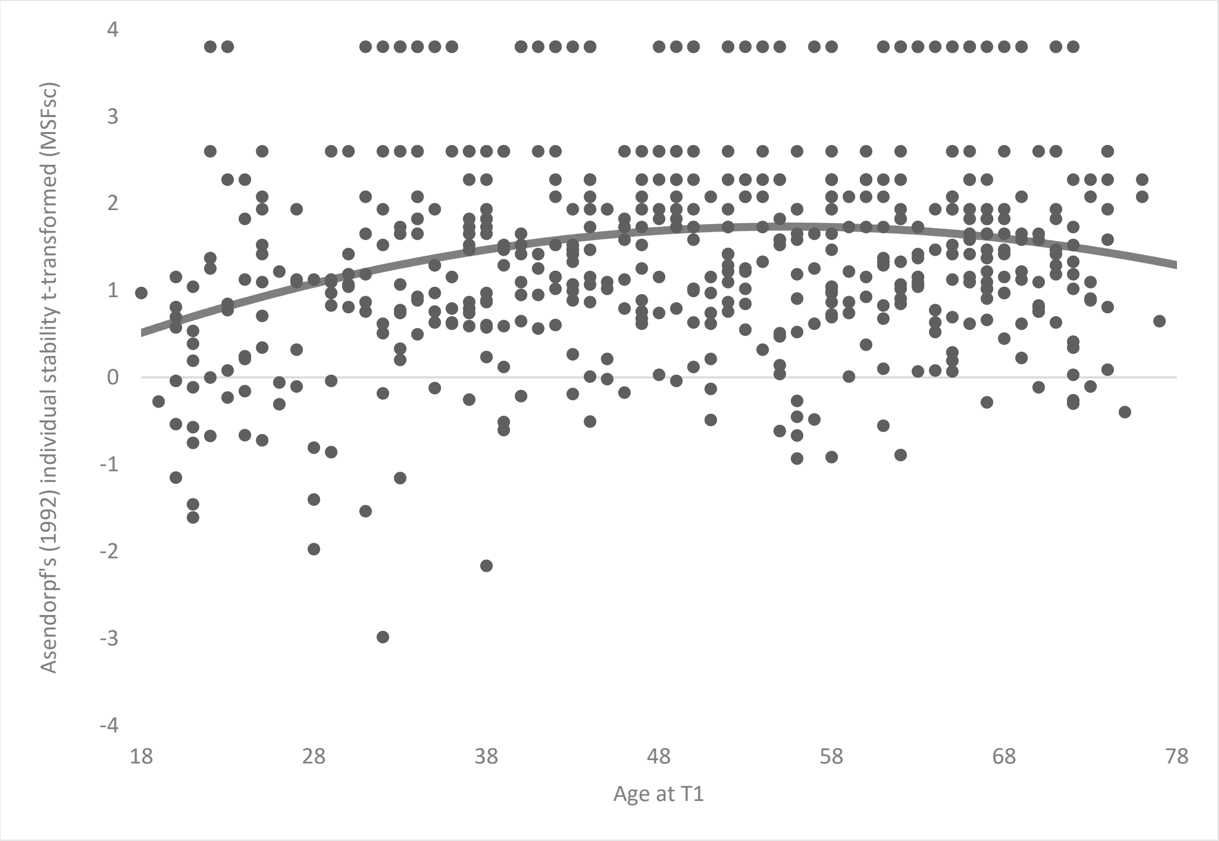

Figure S5.3. *Scatterplot depicting age at T1 on the x-axis and the t-transformed Asendorpf's coefficient of individual stability of MSF*$_{sc}$ *(mid-sleep on free days corrected for sleep debt) on the y-axis.*

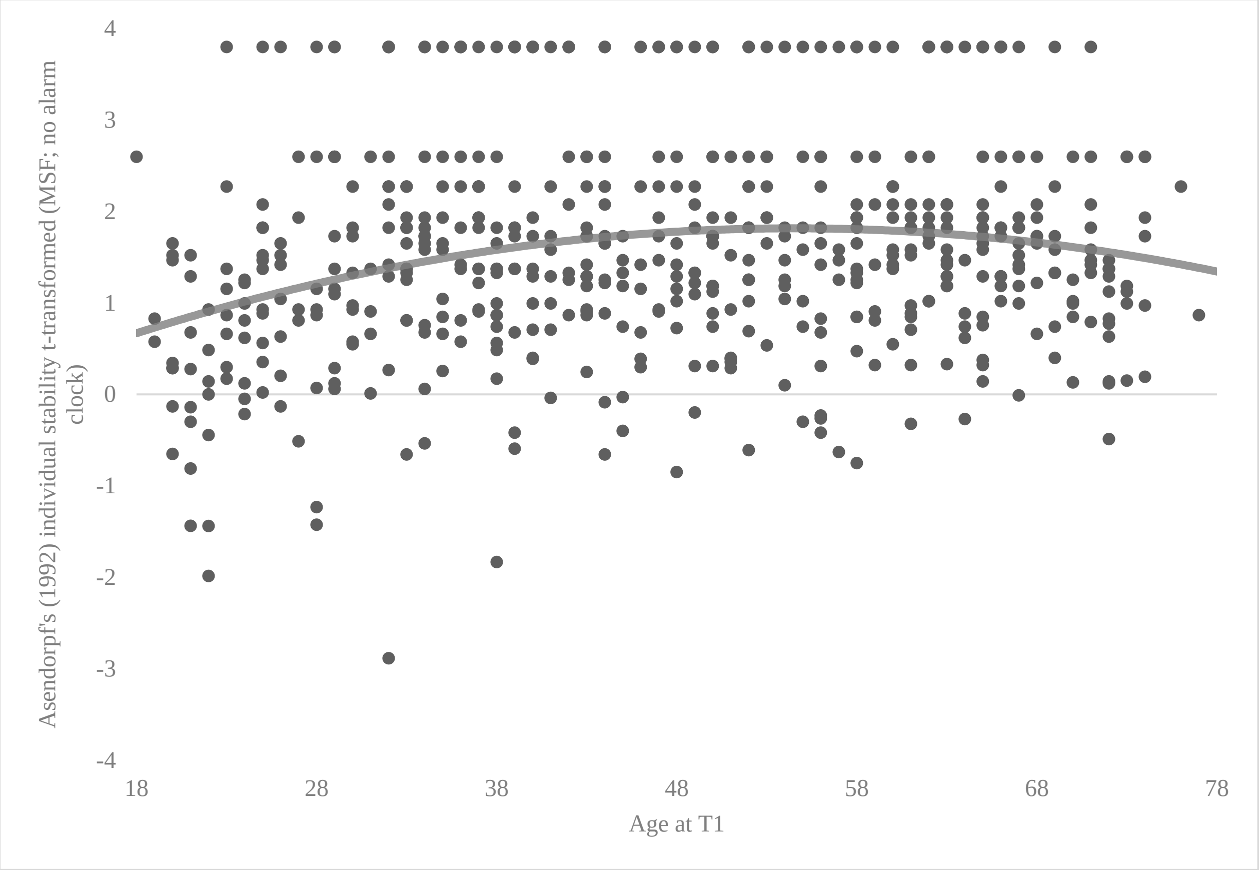

Figure S5.4. *Test-retest correlations of MSF (mid-sleep on free days) and* MSF_{sc} *(mid-sleep on free days corrected for sleep debt) by age at T1.*

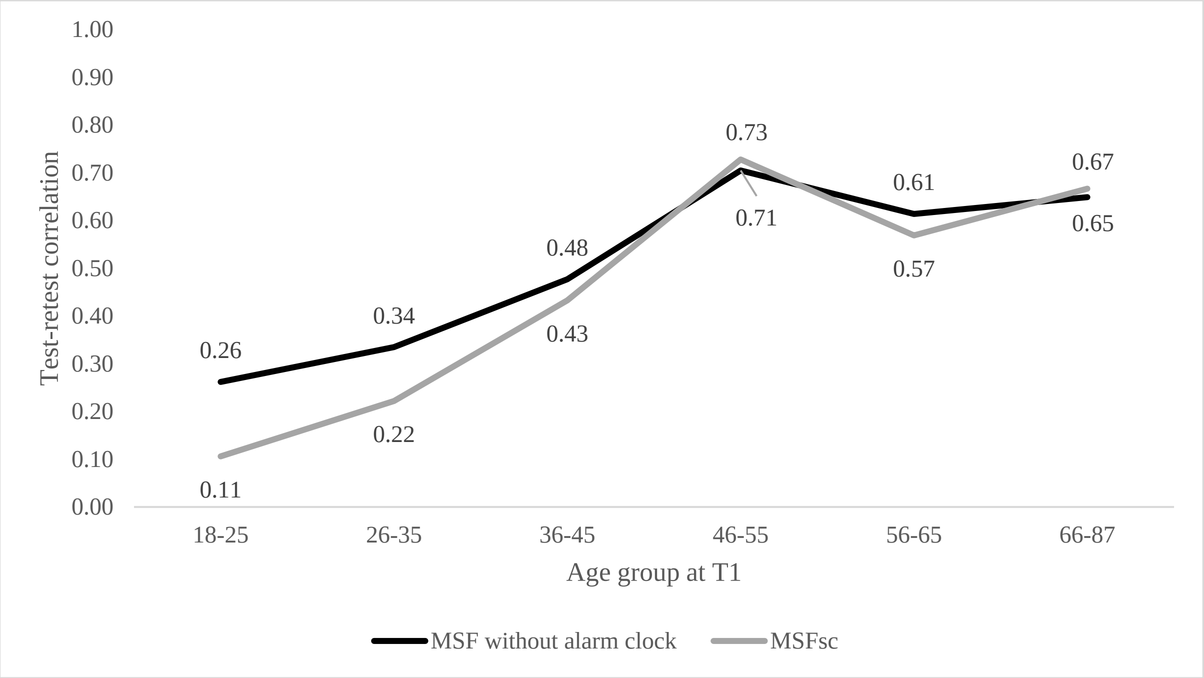

Supplemental Material 6

Figure S6.1. Test-retest correlations of MSF (mid-sleep on free days) and MSW (mid-sleep on workdays) according to the time difference between the two measurements (Study 2).

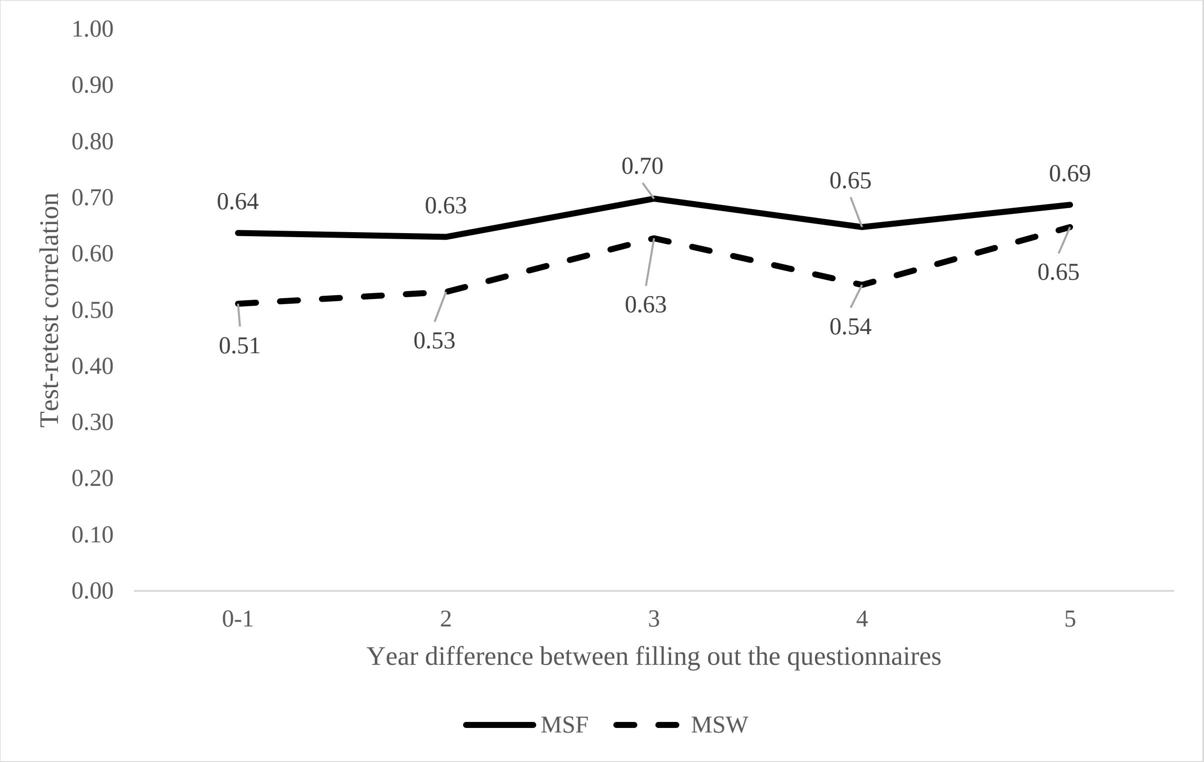

Supplemental Material 7

Figure 7.1. *Scatterplot depicting age at T1 on the x-axis and the t-transformed Asendorpf's coefficient of individual stability of MSF (mid-sleep on free days) on the y-axis (Study 2).*

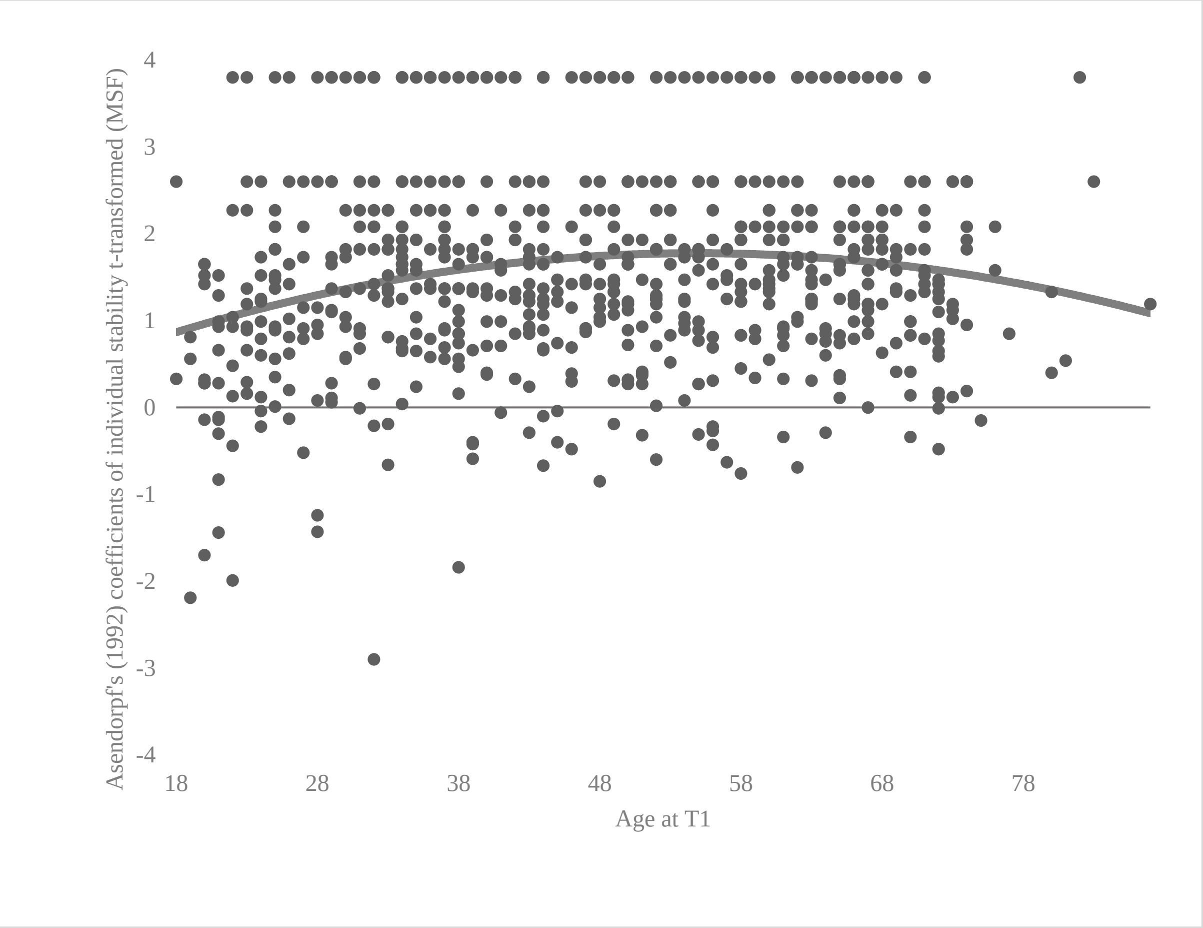

Figure 7.2. *Scatterplot depicting age at T1 on the x-axis and the t-transformed Asendorpf's coefficient of individual stability of MSW (mid-sleep on workdays) on the y-axis (Study 2).*

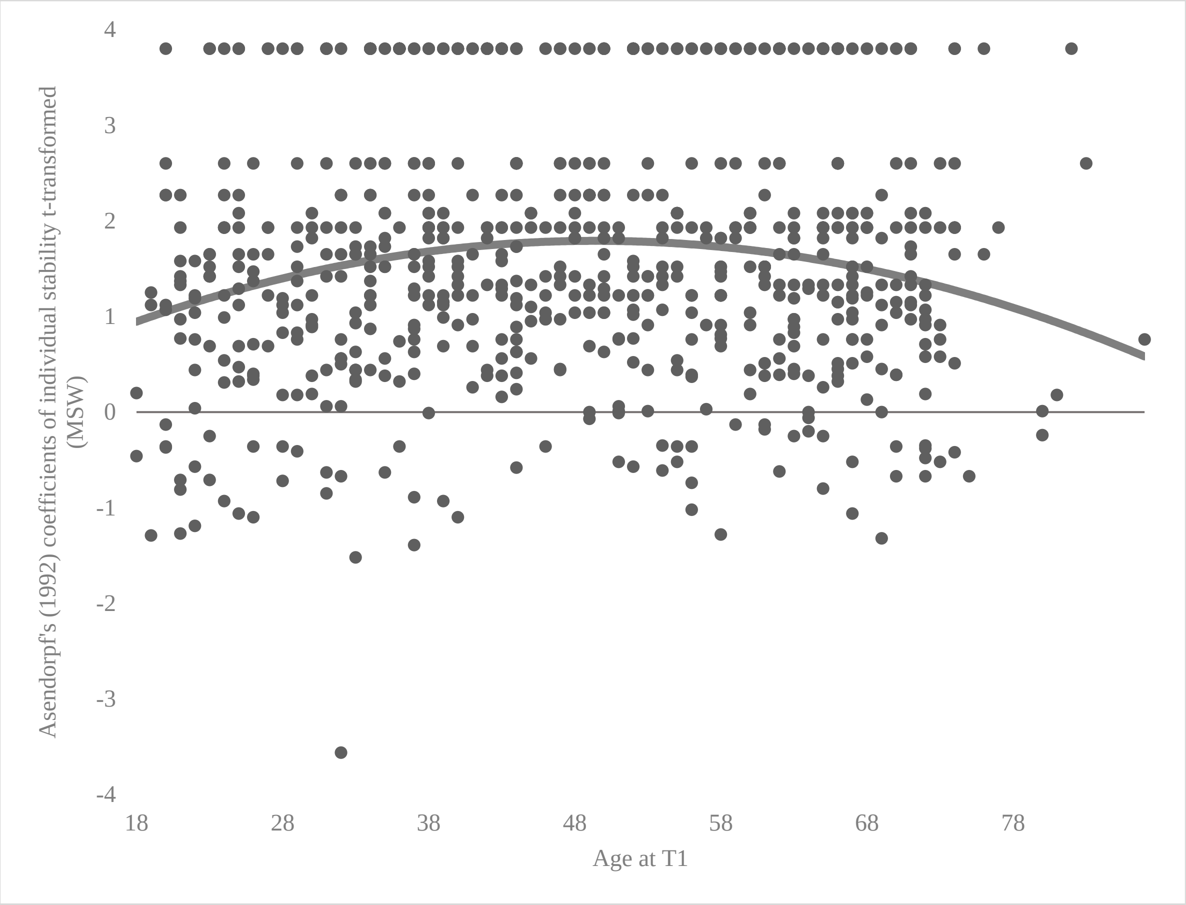

Supplemental Material 8. Hierarchical Multiple Regression Analyses for the Individual Stability of Mid-Sleep on Free Days and Mid-Sleep on Workdays

Table S8.1

Hierarchical Linear Regression Analysis Predicting Asendorpf's (1992) t-Transformed Coefficient of Individual Stability of Mid-Sleep on Free Days (MSF) From Age at T1, its Quadratic Term, and the Difference in Years Between T1 and T2

	Model 1				Model 2				Model 3			
	B [CI]	*β*	*t*	*p*	*B [CI]*	*β*	*t*	*p*	*B [CI]*	*β*	*t*	*p*
**Block 1: Age at T1**												
Constant	1.12 [0.85, 1.40]		7.94	<.001	-0.24 [-1.03, 0.54]		-0.61	.546	-0.04 [-0.09, 0.93]		-.09	.929
Age	0.01 [0.00, 0.02]	.13	3.35	.001	0.07 [0.04, 0.11]	1.00	4.13	<.001	0.08 [0.04, 0.11]	1.02	4.23	<.001
**Block 2: Quadratic age**												
Quadratic term of age					-0.00 [-0.00, 0.00]	-.88	-2.01	<.001	-0.00 [-0.00, 0.00]	-0.90	-3.74	<.001
**Block 3: Time difference**												
Time difference									-0.09 [-0.18, -0.00]	-0.08	-2.00	.045
Adjusted R^2	.015				.032				.037			
F for Change in R^2	11.22			.001	13.31			<.001	4.02			.045
Effect size f^2	.145				.232				.254			

Note. CI = A 95% confidence interval.

Table S8.2

Hierarchical Linear Regression Analysis Predicting Asendorpf's (1992) t-Transformed Coefficient of Individual Stability of Mid-Sleep on Workdays (MSW) From Age at T1, its Quadratic Term, and the Difference in Years Between T1 and T2

	B [CI]	*β*	*t*	*p*	*B [CI]*	*β*	*t*	*p*	*B [CI]*	*β*	*t*	*p*
**Block 1: Age at T1**												
Constant	1.44 [1.13, 1.75]		9.17	<.001	-0.30 [-1.17, 0.57]		-0.68	.498	-0.38 [-1.28, 0.52]		-0.54	.516
Age	0.00 [-.0.00, 0.01]	0.03	0.88	.009	0.09 [0.05, 0.12]	1.04	4.29	<.001	0.08 [0.04, 0.12]	1.03	4.26	<.001
**Block 2: Quadratic age**												
Quadratic term of age					-0.00 [-0.00, 0.00]	-1.02	-4.21	<.001	-0.00 [-0.00, 0.00]	-1.01	-4.17	<.001
**Block 3: Time difference**												
Time difference									.03 [-0.06, 0.13]	0.03	0.71	.477
Adjusted R^2	.000				.024				.023			
F for Change in R^2	0.78			.377	17.69			<.001	0.51			.477
Effect size f^2	0.035				0.195				0.197			

Note. CI = A 95% confidence interval

References

Asendorpf JB (1992) Beyond stability: Predicting inter-individual differences in intra-individual change. Eur J Pers 6:103-117.

Caci H, Nadalet L, Staccini P, Myquel M, and Boyer P (2000) The Composite Scale of Morningness: Further psychometric properties and temporal stability. Eur Psychiatry 15:278-281.

Druiven SJM, Hovenkamp-Hermelink JHM, Knapen SE, Kamphuis J, Haarman BCM, Penninx B, Antypa N, Meesters Y, Schoevers RA, and Riese H (2020) Stability of chronotype over a 7-year follow-up period and its association with severity of depressive and anxiety symptoms. Depress Anxiety 37:466-474.

Greenwood KM (1994) Long-term stability and psychometric properties of the Composite Scale of Morningness. Ergonomics 37:377-383.

Griefahn B, Künemunnd C, Bröde P, and Mehnert P (2001) Zur Validität der deutschen Übersetzung des Morningness-Eveningness-Questionnaires von Horne und Östberg. [The validity of a German Version of the Morningness-Eveningness-Questionnaire developed by Horne and Östberg]. Somnologie 5:71-80.

Horne JA, and Östberg O (1976) A self-assessment questionnaire to determine morningness-eveningness in human circadian rhythms. Int J Chronobiol 4:97-110.

Kantermann T, and Eastman CI (2018) Circadian phase, circadian period and chronotype are reproducible over months. Chronobiol Int 35:280-288.

Kühnle T (2006) Quantitative Analysis of Human Chronotypes [PhD Thesis]. [Munich]: Ludwig-Maximilians-Universität München.

Larsen RJ (1985) Individual differences in circadian activity rhythm and personality. Pers Individ Dif 6:305-311.

Lee JH, Kim SJ, Lee SY, Jang KH, Kim IS, and Duffy JF (2014) Reliability and validity of the Korean version of Morningness-Eveningness Questionnaire in adults aged 20-39 years. Chronobiol Int 31:479-486.

McMahon DM, Burch JB, Wirth MD, Youngstedt SD, Hardin JW, Hurley TG, Blair SN, Hand GA, Shook RP, Drenowatz C, Burgess S, and Hebert JR (2018) Persistence of social jetlag and sleep disruption in healthy young adults. Chronobiol Int 35:312-328.

Neubauer A (1992) Psychometric comparison of two circadian rhythm questionnaires and their relationship with personality. Pers Individ Dif 13:125-131.

Pündük Z, Gür H, and Ercan İ (2005) A reliability study of the Turkish version of the Morningness-Eveningness Questionnaire. Turkish Journal of Psychiatry 16:i-vi.

Reis C, Madeira SG, Lopes LV, Paiva T, and Roenneberg T (2020) Validation of the Portuguese variant of the Munich Chronotype Questionnaire ($MCTQ^{PT}$). Front Physiol 11:1-10.

Roenneberg T (2015) Having trouble typing? What on earth is chronotype? J Biol Rhythms 30:487-491.

Roenneberg T, Wirz-Justice A, and Merrow M (2003) Life between clocks: Daily temporal patterns of human chronotypes. J Biol Rhythms 18:80-90.

Smith CS, Reilly C, and Midkiff K (1989) Evaluation of three circadian rhythm questionnaires with suggestions for an improved measure of morningness. J Appl Psychol 74:728-738.

Suh S, Kim SH, Ryu H, Choi SJ, and Joo EY (2018) Validation of the Korean Munich Chronotype Questionnaire. Sleep Breath 22:773-779.

Urner M, Tornic J, and Bloch KE (2009) Sleep patterns in high school and university students: A longitudinal study. Chronobiol Int 26:1222-1234.

Wood J, Birmaher B, Axelson D, Ehmann M, Kalas C, Monk K, Turkin S, Kupfer DJ, Brent D, Monk TH, and Nimgainkar VL (2009) Replicable differences in preferred circadian phase between bipolar disorder patients and control individuals. Psychiatry Res 166:201-209.
